# Bifunctional Liquid Metals Allow Electrical Insulating Phase Change Materials to Dual-Mode Thermal Manage the Li-Ion Batteries

**DOI:** 10.1007/s40820-022-00947-w

**Published:** 2022-10-10

**Authors:** Cong Guo, Lu He, Yihang Yao, Weizhi Lin, Yongzheng Zhang, Qin Zhang, Kai Wu, Qiang Fu

**Affiliations:** 1 College of Polymer Science and Engineering, State Key Laboratory of Polymer Materials Engineering, Sichuan University, Chengdu, 610065 People’s Republic of China; 2 Department of Polymer Science and Engineering, School of Chemical Engineering, Nanjing University of Science and Technology, Nanjing, 210094 People’s Republic of China

**Keywords:** Phase change materials, Liquid metal, Thermal conductivity, Photothermal conversion, Battery thermal management

## Abstract

Phase change materials (PCMs) are expected to achieve dual-mode thermal management for heating and cooling Li-ion batteries (LIBs) according to real-time thermal conditions, guaranteeing the reliable operation of LIBs in both cold and hot environments. Herein, we report a liquid metal (LM) modified polyethylene glycol/LM/boron nitride PCM, capable of dual-mode thermal managing the LIBs through photothermal effect and passive thermal conduction. Its geometrical conformation and thermal pathways fabricated through ice-template strategy are conformable to the LIB’s structure and heat-conduction characteristic. Typically, soft and deformable LMs are modified on the boron nitride surface, serving as thermal bridges to reduce the contact thermal resistance among adjacent fillers to realize high thermal conductivity of 8.8 and 7.6 W m^−1^ K^−1^ in the vertical and in-plane directions, respectively. In addition, LM with excellent photothermal performance provides the PCM with efficient battery heating capability if employing a controllable lighting system. As a proof-of-concept, this PCM is manifested to heat battery to an appropriate temperature range in a cold environment and lower the working temperature of the LIBs by more than 10 °C at high charging/discharging rate, opening opportunities for LIBs with durable working performance and evitable risk of thermal runaway.

**Supplementary Information:**

The online version contains supplementary material available at 10.1007/s40820-022-00947-w.

## Highlights

- The phase change materials possess conformable configuration to the structure of Li-ion batteries in macro-scale and multidirectional thermal pathways for rapid and uniform heat transfer in micro-scale.
- Hierarchically structured phase change materials achieve dual-mode thermal management ability for heating and cooling Li-ion batteries.
- Excellent practical battery thermal management performance was verified by 18,650 Li-ion batteries in both cold and hot environments.

**Supplementary Information:**

The online version contains supplementary material available at 10.1007/s40820-022-00947-w.

## Graphical abstract

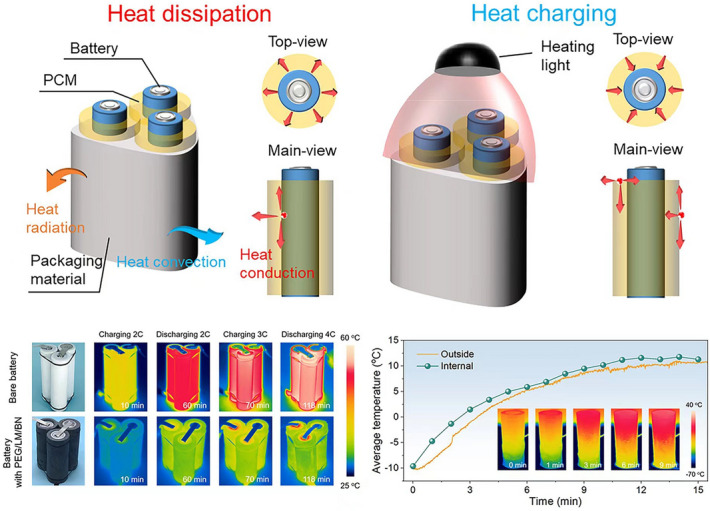

## Introduction

**Fig. 1:**
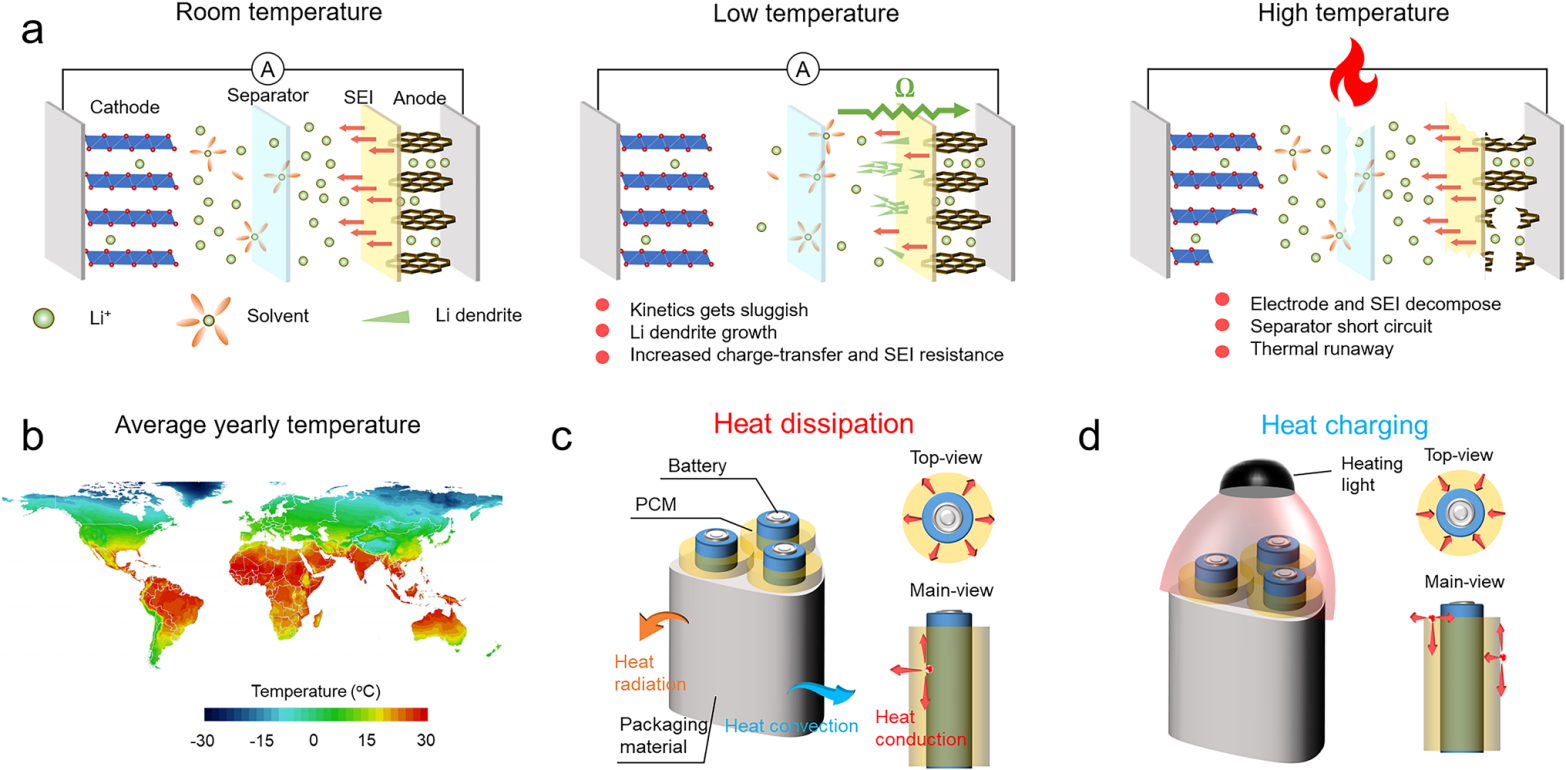
**a** Mechanisms of battery performance degradation at low temperature and high temperature from a material perspective. **b** Geographic distribution of world average annual temperature [12]. **c** Working principle of passive battery thermal management at high temperature using our PCM thermal regulator. **d** Working principle of heating of battery in a cold environment through light-to-heat conversion

To curb greenhouse gas emissions and suppress the dependence on traditional fossil energy, Li-ion batteries (LIBs) combining high energy density and light weight are developed and now play prominent parts in the rechargeable applications like consumer electronics, electric vehicles and energy storage equipment [1–3]. Notably, degraded operation performance, reduced life span and safety problems of LIBs would be caused by low or high temperature. For example, it would happen in the low temperature (below 0 °C) that battery kinetics process gets more sluggish, resulting in capacity loss and increased internal resistance, and the growth of dendritic Li planting compromises the lifetime and battery safety (Fig. 1a) [4–6]. High temperature contributes to irreversible capacity loss and electrode degradations that accelerate battery aging, and continuously rising temperature further triggers internal short circuits and catastrophic thermal runaways, posing threats to the battery property and humankind’s safety [6–11]. On a global scale, the high-latitude country area has an average yearly temperature below 0 °C, while the tropical zone commonly suffers from hot weather (Fig. 1b) [12]. Therefore, it is necessary to achieve dual-mode thermal management for heating or cooling LIBs according to real-time thermal conditions to maintain the performance of LIBs within a wider temperature range and expand their usability [13].

Phase change materials (PCMs) with high thermal conductivity (*k*) and latent heat are regarded as important passive thermal management materials in the application of LIBs, which could dissipate the accumulated heat inside the LIBs rapidly to realize temperature uniformity and present a buffer against temperature rise [14–19]. Previous PCMs were usually prepared by incorporating thermo-conductive fillers into the PCM matrix, paying tremendous attention to the *k* enhancement [20–23]. For example, the methods including the construction of an interconnected filler network, the orientation of anisotropic filler, and the interfacial enhancement between filler and matrix were adopted to improve the *k* of PCMs [24–36]. However, *k* determines the thermal conduction rate within a PCM in one specific direction; in addition to *k*, the thermal conduction directions and paths are identically important. It is considered that exquisite structural manipulations need to be proposed to regulate the thermal conduction directions and paths, leading which to be conformable to the macroscopical structure and heat-conduction characteristic of the LIBs [37]. For example, individual batteries generally have a cylindrical appearance, whose heat generations and thermal runaways are usually reflected by localized hot spots. This indicates that future PCMs for the thermal management of LIBs should conform to the cylindrical configuration and their heat-conduction characteristic should meet the radial and through-plane heat dissipation requirements. Moreover, PCMs are also required in cold environments to provide sufficient heating function through some effects like electrothermal effect, photothermal effect or magnetocaloric effect. However, such PCMs with dual-mode thermal management ability have never been previously studied and reported.

Herein, taking advantage of dual thermal effects (high thermal conductivity and prominent photothermal conversion) of liquid metal (LM), we report an elaborate polyethylene glycol (PEG)/LM/boron nitride (BN) PCM, which could arbitrarily manipulate battery temperature in both hot and cold extreme environments [38–41]. On the one hand, to realize rapid and uniform passive cooling at high temperature, the multi-directional thermal pathways were constructed by a radial ice-template assembly of LM modified BN in a concentric-rings mold, followed by an infiltration of the phase-change PEG. This was designed to conform to the uneven calorification characteristic of LIBs and meet the requirement of isotropic heat transfer, enabling localized heat to spread to the surroundings rapidly. Different from incompact contact of rigid fillers, deformable LM droplets coated on the surface of the contiguous BN sheets could fuse with each other to reduce the interfacial thermal resistance, yielding a through-plane thermal conductivity (*k*_⊥_) as high as 8.8 W m^−1^ K^−1^ and an in-plane thermal conductivity (*k*_//_) of 7.6 W m^−1^ K^−1^. As a result, the PCM successfully lowered the maximum temperature of the commercial 18,650 LIBs by more than 10 °C at high charging/discharging rate and relieved the battery capacity loss under extremely fast-charge conditions, contributing to the device safety and prolonged lifetime. On the other hand, when LIBs face low temperature ambient, a battery thermal management system was equipped with advanced temperature sensor and laser transmitter. The PCM could pre-heat the battery cells from sub-zero temperature to an appropriate range above freezing by virtue of the excellent photothermal conversion attributed to the plasmonic effect of LM. Along with other advantages like remarkable phase change figure of merits (1162.7 × 10^6^ W^2^ S/m^4^ °C), shape stability, electrical insulation, flame resistance and cycling durability, this concept of PCMs achieving dual-mode thermal regulator of LIBs is believed to be promising for energy conservation and energy security in the sustainable development of modern life.

## Experimental Section

### Materials

Liquid metal (Galinstan) was provided by Zhenjiang Fanyada Electronic Technology Co., Ltd. Hexagonal boron nitrogen (BN) powders with different lateral sizes were obtained from Qinhuangdao Eno High-Tech Material Development Co., Ltd. (China). Sodium carboxymethylcellulose (SCMC, viscosity: 1000–1400 mPa s) and Polyethylene glycol (PEG, *M*_n_ = 4000) was purchased from Aladdin. All chemicals were directly used without further purification. The commercial 18,650 LIBs (AB3, nominal voltage of 3.7 V, charge voltage of 4.2 V, nominal capacity of 2300 mAh, anode of graphite, cathode of lithium cobaltate) was phased from the brand SUPFIRE.

### Preparation of LM/BN Skeleton

The LM/BN hybrid fillers were first prepared by mixing various loading of liquid metal with the micrometer-sized BN powders, followed by ball-milling treatment for 20 min. The collected LM/BN hybrids were subsequently dispersed into 1 wt% SCMC deionized water solution which was used as a dispersion agent and molecular linker. The mixture was ball milled (MQ-3SP2) for 2 h. To construct the radially oriented skeleton, the LM/BN suspension were poured into a customized mold, which is consist of a copper hollow cirque (34 mm outside diameter, 28 mm inner diameter, 72 mm height), two polytetrafluoroethylene (PTFE) cylindrical molds (one with 18 mm diameter and 67 mm height as thermal insulator in the center, another with 28 mm diameter and 5 mm height as pedestal). The whole mold was put on a solid copper cylinder (38 mm diameter, 150 height) which was immersed in liquid nitrogen for 30 min. Then the frozen slurries were put into the freeze-dryer for more than 48 h so that the radially oriented LM/BN skeleton was obtained. As the contrast sample, the radial BN skeleton was constructed by employing the pristine BN powders without modification to perform the identical fabrication process.

### Fabrication of PEG/LM/BN Phase Change Composites

The composites were prepared via vacuum-assist infiltration of PEG into LM/BN skeleton. First, PEG was melted in a vacuum drying oven at 80 °C. When air bubbles were no longer produced, the LM/BN skeleton was put into the melted PEG. After that, by continuously degassing every 5 min at 70 °C, PEG was infiltrated into LM/BN skeleton slowly. The process had to continue for at least 6 h and then the composites were solidified below their melting temperature. The same vacuum-assist infiltration of PEG into BN skeleton enabled the PEG/BN composites.

### Characterization

The microscopic morphology and structure were characterized by a high-resolution field emission scanning electron microscope (SEM, Apreo S, USA) and an Atomic Force Microscope (AFM, Bruker Icon, USA). The interior structure was investigated by a Bruker Skyscan1272 Micro computed tomography (micro-CT) with resolution of 1 μm. The elemental states of BN and LM/BN powder were characterized by X-ray photoelectron spectroscopy (XPS, Thermo K-Alpha, USA). Density functional theory (DFT) simulation was conducted by using Materials Studio 2017 R2. In-plane and out-of-plane thermal conductivity were calculated by the equation *k* = *α* × *ρ* × *C*_p_ (*α*: the thermal diffusivity, *ρ*: the density, *C*_p_: the specific heat capacity). Laser flash analysis (LFA 467 Hyper Flash, Netzsch, Germany) was used to measure *α* of samples. Both sides of the sample were sprayed with graphite in order to guarantee the identical absorption of laser energy. *C*_p_ can be determined by differential scanning calorimetry (STA 449F3, Germany) with sapphire as the standard sample. *C*_p_ of the composite can be calculated by the equation *C*_p-composite_ = *C*_p-A_ *φ* + *C*_p-B_ (1−*φ*), where *C*_p-A_ is the specific heat capacity of constituent A, *C*_p-B_ is the specific heat capacity of constituent B, and *φ* is the mass ratio of constituent A. The sample density (*ρ*) was measured by using the Archimedes method (JA203 Puchun Instruments, China). Differential scanning calorimetry (DSC 8000, PerkinElmer, USA) was used to test the thermal storage performance and verify the thermal stability at a scanning rate of 10 °C min^−1^. The UV–vis-NIR spectra were obtained through an ultraviolet–visible near-infrared spectrophotometer (UV3600, Shimadzu, Japan) while the light absorption (A) can be calculate using the formula of A = 1-R-T, where R means reflectance and T means transmittance. To evaluate the photothermal performance, a tailor-made foam chamber was used to not only fix the PEG/LM/BN composite with the highest *k* but also hold liquid nitrogen used to simulate a subzero environment, enabling the PEG/LM/BN composite to be over the liquid nitrogen. The testing sample was irradiated by a laser (532 nm) and the temperature trend of the opposite surface was recorded by an infrared thermal camera (FLIR-T600) to visualize the photothermal conversion process. To investigate the heat dissipation capacity of the samples, a powerful ceramic heater (Ф 5 mm, 3.7 V, 12 W) was attached to the center of samples, while the temperature was recorded by an infrared camera (FLIR-T600). A Xenon lamp (CEL-S500) was used to irradiate the sample to achieve photothermal conversion of the thermal regulator, while it was cooled by liquid nitrogen at the same time. The thermocouple (with 2 mm diameter and 0.3 mm long) used in this work for surface temperature measurement is T-type and meets the criterion of ANSI MC96.1. The operating current, voltage and capacitance were performed on a charge–discharge balancer (HOTA D6).

## Results and Discussion

### Structural Design and Preparation of the PEG/LM/BN PCM

By using a radial ice-template assembly method in a customized mold, the resultant PCMs are expected to exhibit a typical hierarchical structure. The construction in macro-scale is conformable to the shape and structure of the commercial 18,650 LIBs, and the micro-scale interconnected LM/BN hybrid fillers are tailored to enable the continuous and multi-directional thermal pathways adapted for the heat-production characteristic of LIBs. With the hierarchical structure, our PCM is possible to achieve dual-mode thermal management ability for heating and cooling Li-ion batteries by the bifunctions of LM through the passive thermal conduction and photothermal effect (Fig. 1c, d).

**Fig. 2:**
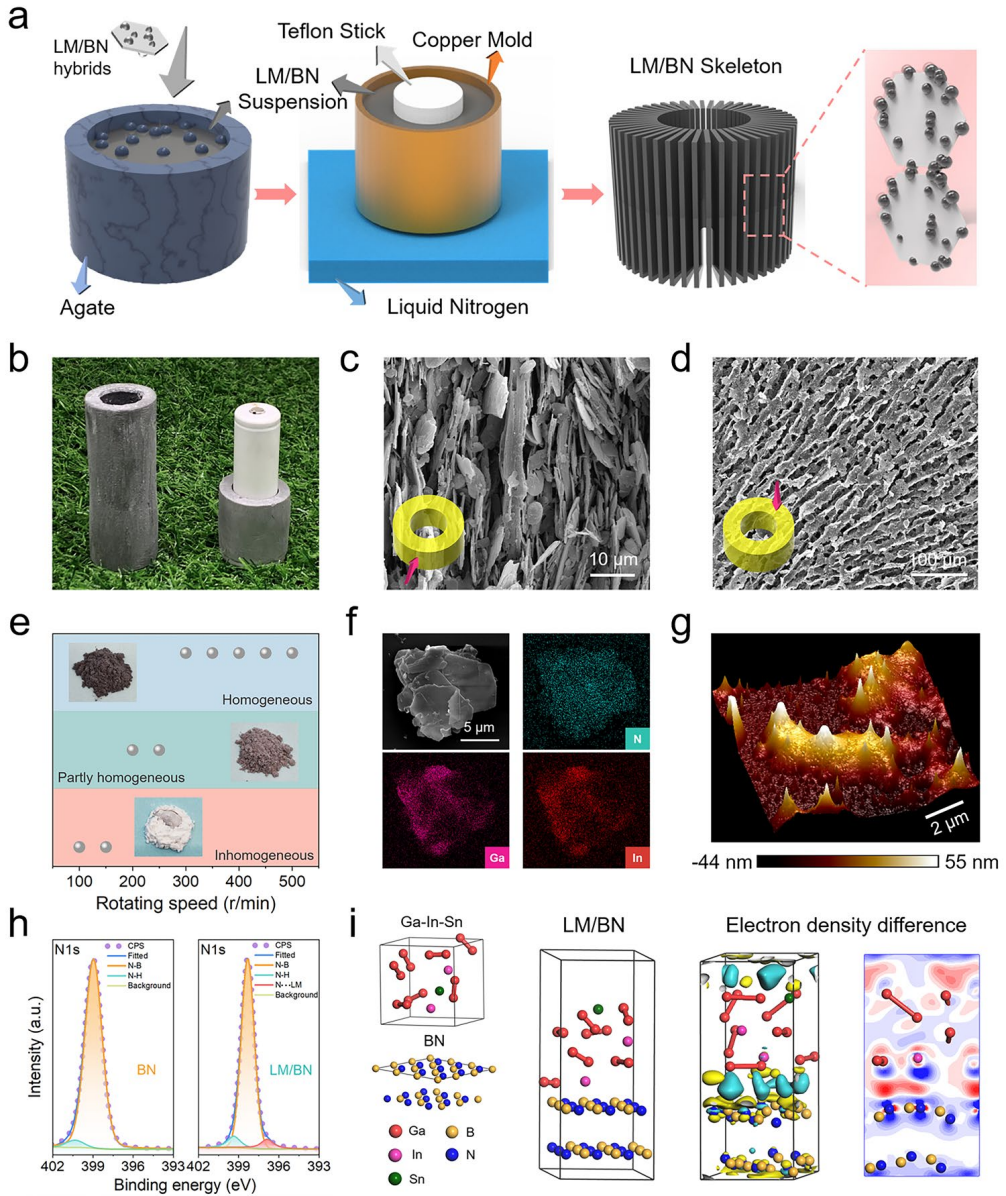
**a** Schematic of the preparation process for the LM/BN skeleton with radially oriented thermal pathways. **b** Digital photograph of the PEG/LM/BN PCM thermal regulator which is conformable to the structure of a 18,650 LIB. **c** Scanning electronic macroscopy (SEM) images showing longitudinal and **d** cross-sectional morphology of the thermal regulator made of PEG/LM/BN composite. **e** Morphological variation of LM/BN binary hybrids with the increasement of ball-milling speed which could offer larger mechanical force. The pink region manifests the phase separation. The green region manifests the formation of the partly homogeneous phase. The blue region indicates the complete formation of the homogeneous phase. **f** SEM element mapping result of the LM/BN hybrid filler proving successful modification of the pure BN sheets by LM. **g** Three-dimensional height image of the LM/BN hybrid filler from atomic force microscopy result. **h** X-ray photoelectron spectroscopy N 1*s* spectra of BN (left) and LM/BN hybrid filler (right). **i** Density functional theory models and difference charge density image of BN approaching LM. In the three-dimensional image, the yellow region represents electron loss and the blue color represents electron aggregation. The two-dimensional image is from a cross section of the three-dimensional image and the blue color represents electron loss and the red color represents electron aggregation

The detailed fabrication process could be seen in the experimental section. Specially, to construct the LM/BN skeleton, we adopted a structured copper mold with adiabatic Teflon stick in the center, inducing the alignments of ice crystals along the temperature gradients including radial direction and bottom-up direction once the mold was immersed in liquid nitrogen (Figs. 2a and S1) [42]. Additionally, we could change the dimension of the Teflon stick to match different types of batteries, which provides manufacturing flexibility for practical application. After impregnating LM/BN skeleton with PEG, a finished PCM-based thermal regulator adapted for the shape of commercial LIB was successfully fabricated, as shown in Fig. 2b. The specific structural morphology of the thermal regulator was characterized by SEM images. Figure 2c, d shows overlapped and bottom-up orientation in the longitudinal view and long-range interconnected and radially alignment in the cross-sectional view of LM/BN inclusions, contributing to multi-directional and continuous BN-BN thermal pathway. Simultaneously, BN sheets act as carriers of LM droplets, thus well-aligned BN network enables uniform distribution of thermogenic LM components within the PCM, which could be observed through micro computed tomography (CT) images (Fig. S2).

BN, a typical two-dimensional material with intrinsic high *k*_//_ (theoretical value could be more than 600 W m^−1^ K^−1^), was commonly selected as basic unit to fabricate thermally conductive but electrically insulating materials [43, 44]. However, due to its wide bandgap, BN with white color fails to exhibit optical absorption. Different from conventional studies, we enabled BN to be coated by LM through a mechanochemistry approach to obtain LM/BN hybrid fillers. For LM as modifier, there are two thermal effects: (i) LM integrating liquid-like fluidity and metal-level *k* (up to 39 W m^−1^ K^−1^) could interfacially merge together without stereo mismatch like the contact of the rigid fillers to reduce contact thermal resistance among adjacent fillers [45]; (ii) excellent photothermal conversion ability of LM uniformly embedded in composites endows the resultant thermal regulator with light-stimulated heat-generation resonance [41, 46].

LM/BN binary hybrid fillers were employed as basic thermoconductive units, thus the successful preparation of them is an essential step in this work. The LMs we chose were eutectic alloys of Galinstan whose melting temperature was 13.2 °C, which present deformability under room temperature and tremendous mismatch in surface energy compared to solid BN sheets. To enable LM to be stably anchored to the surface of BN platelets, we took advantage of mechanical ball-milling to conduct the mechanochemistry reaction [47]. Unlike previously reported modification method based on traditional chemistry grafting, the mechanochemistry procedure was simple, time-saving and high-yield to obtain the thermally conductive hybrid fillers. The effect of mechanical force on the modification of LM was studied by adjusting the rolling speed, and with the increase in ball-milling speed, the entirely inhomogeneous mixture of white BN powder and LM droplets with a phase-separate could be turned into gray homogeneous LM/BN hybrid fillers at 300 rpm and above, manifesting complete reaction between LM and BN (Fig. 2e). The SEM and the corresponding elemental mapping result demonstrate that LM has been firmly coated on the surface of BN platelets (Fig. 2f) and AFM image further clearly reflects the island-shaped distribution of LM droplets on the surface of a single BN sheet through height variation and phase diversity (Figs. 2g and S3). Moreover, XPS analysis was performed to study the interaction between LM and BN which are thermodynamically incompatible (Fig. 2h). It is found that an additional deconvolved peak at ~ 397 eV occurs in the N 1*s* peak image of LM/BN hybrid filler compared to that of the pristine BN, which may be assigned to the coordination bonding generated during mechanochemistry reaction process. In Fig. 2i, DFT simulation further verifies the formation of the coordination. In the resultant two-dimensional difference charge density image, which is from a cross section of the three-dimensional image, the blue color represents electron loss and the red color represents electron aggregation, thus a distinct electron shift from N atoms toward Ga atoms could be found when Ga–In–Sn alloys approach the surface of BN crystals, indicating the lone pair of electrons outside the N atoms enter the empty orbitals of Ga atoms at the heterogeneous interface.

### Thermal Conductivities and Phase-Change Properties of the PEG/LM/BN Composites

**Fig. 3:**
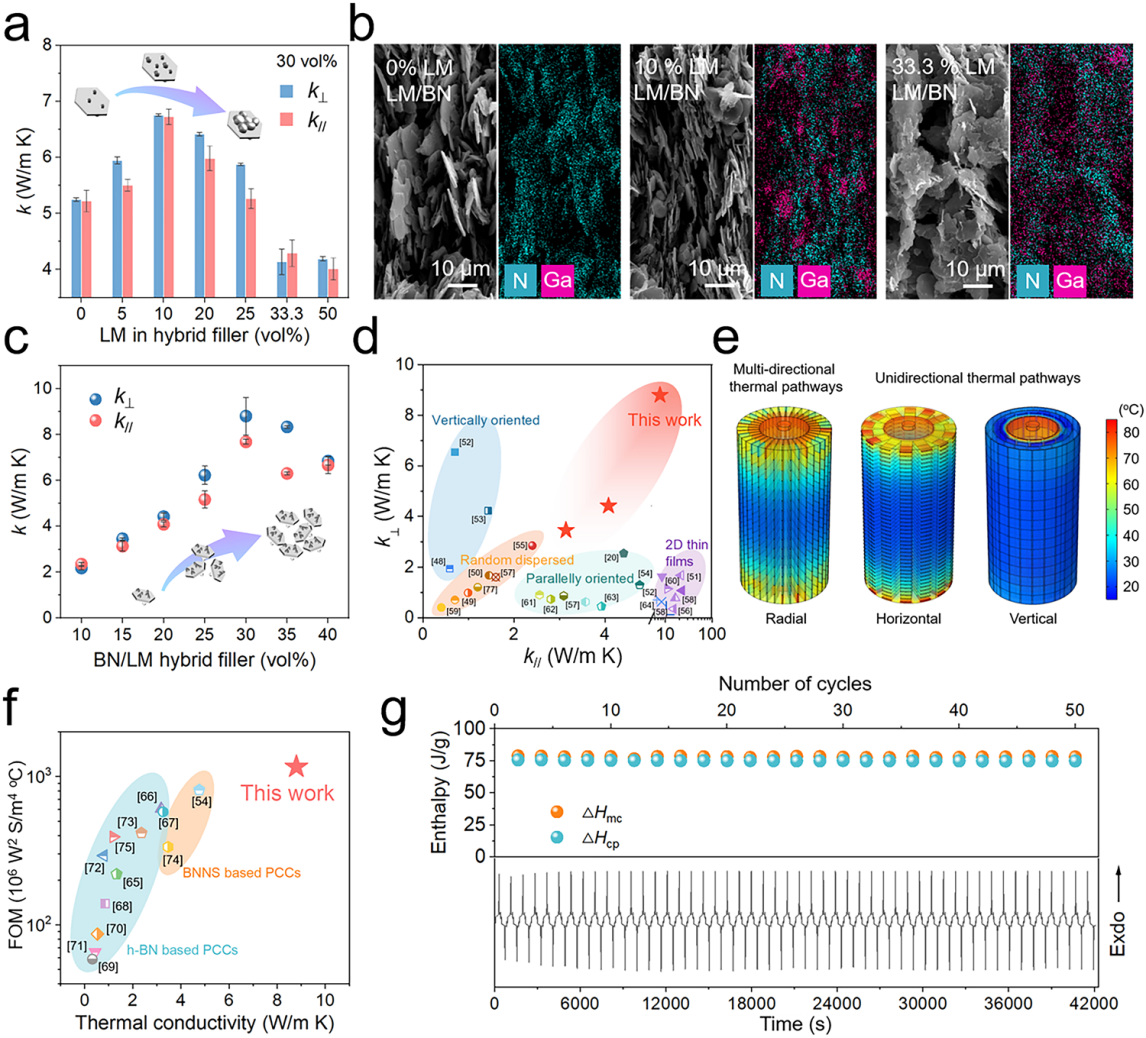
**a** *k*_⊥_ and *k*_//_ of the PEG/LM/BN composites as a function of the LM content in hybrid fillers (~ 10 μm). Note that the total volume fraction of LM/BN hybrid fillers in composites is a constant value of 30%. **b** SEM images taken from the longitudinal section and the corresponding nitrogen and gallium element mapping of the PEG/LM/BN composites incorporating 30 vol% LM/BN hybrid fillers (~ 10 μm) with LM content of 0, 10 and 33.3 vol%, respectively. **c** *k*_⊥_ and *k*_//_ of the PEG/LM/BN composites as a function of the volume fraction of the binary LM/BN hybrid fillers (~ 18 μm). **d** Comparison of *k*_⊥_ and *k*_//_ of our PEG/LM/BN composites with previously reported BN-based polymer composites [48–64]. **e** Direct temperature distribution of simulated model of PEG/LM/BN composites with radial, horizontal and vertical orientations which are used to carry out battery thermal management, calculated by finite element simulation (FES). **f** Comparison of the figure of merits and *k* of BN-based phase change composites from the latest studies [54, 65–75]. **g** Calculated enthalpy of melting and crystallization (top) and the differential scanning calorimetry profiles (bottom) of the PEG/LM/BN composite with the highest *k* for 50 cycles

Laser flash analysis was carried out to investigate the thermally conductive properties of the composites. We employed the pristine BN (~ 10 μm) and the binary hybrid fillers with different LM content (Fig. S4) to fabricate the composites with the identical filler volume fraction (30 vol%), investigating the role of LM in *k* enhancement. LM in hybrid filler of 10 vol% enabled the composite to reach the highest isotropic *k* (Fig. 3a). At the same time the composites were maintained in the scope of electrical insulator due to high intrinsic electrical resistance of BN and disconnected electrically percolative network of LM droplets (Fig. S5a). In Fig. 3b, from the SEM images and the corresponding elemental mappings of the longitudinal section, it could be found that there are still interfacial defects within the vertically oriented thermal pathways constructed by BN sheets without modification of LM. Therefore, the phonon scattering at the interface could impede the heat flux within the composites. However, a small amount of LM coated on the surface of BN sheets tended to merge together to occupy the regions among the adjacent hybrid fillers during the process of fillers orientation driven by ice crystals growth, promoting the formation of more continuous thermal pathways. This bridge effect could be also clearly verified by the backscatter electron (BSE) image (Fig. S6). With the proportion of LM in hybrid fillers increasing, the degree of orientation of hybrid fillers along vertical direction declines owing to the higher density of the LM/BN inclusions, accounting for the downtrend of the thermal conductivity of the PEG/LM/BN composites. Then, the identical experiment was conducted by utilizing BN sheets with a lateral size of ~ 18 μm, likewise proving LM in hybrid filler of 10 vol% is the optimized modification ratio for *k* enhancement (Fig. S7).

Based on the result, LM/BN hybrid fillers with different lateral sizes and the same LM modification content (10 vol%) were employed to study the size effect on *k* of composites (Fig. S8), as the filler volume fractions of the fabricated composites were maintained at 30 vol%. Consequently, the lateral size of ~ 18 μm is the optimization of enhancing the *k*. Furthermore, the *k* of the PEG/LM/BN composites embedded with 10–40 vol% LM/BN binary hybrid fillers with lateral size of ~ 18 μm and LM modification content of 10 vol% are plotted in Fig. 3c. Increasing introduction of LM/BN hybrids contributes to the formation of more thermally conductive pathways in the composites, whereas the higher viscosity originating from increasing loading of hybrid fillers deteriorates the orientation of LM/BN hybrids. When loaded with 30 vol% hybrid fillers, the composite exhibits the highest *k*_⊥_ of 8.8 W m^−1^ K^−1^, which is 29.3-fold that of pure PEG (0.3 W m^−1^ K^−1^). Meanwhile, *k*_//_ of 7.6 W m^−1^ K^−1^ reveals the isotropic thermoconductive capability of PEG/LM/BN composites with radial orientation. Despite high filler content, the composites still maintain electrically insulating (Fig. S5b). Compared with reported BN-based polymer composites with fillers dispersed randomly or aligned along unidirectional direction, our composites achieve high *k* in both through-plane and in-plane direction, filling the vacancy for polymer composites (Fig. 3d and Table S3) [48–64]. This could be attributed to sophisticated design of multi-directional thermal pathways by leveraging the advantages of BN’s hexagonal dimension and ultrahigh *k*_//_, and reduced interface thermal resistance by LM’s interfacial fusion. For the former, finite element simulation (FES) was carried out to visualize the superiority of multi-directional thermal pathways in rapid and uniform thermal transmission (as shown in Figs. 3e and S9). Specifically, when local overheating occurs in electrodes, the surface temperature of the thermal regulator with multi-directional thermal pathways grows much faster than that with merely horizontal or vertical thermal pathways with heating time increasing, suggesting its higher speed rate of cell-to-air heat transfer. In addition, we found the maximum temperature difference along z-axis on the outer surface of horizontally oriented thermal regulator is much higher than that of radial thermal regulator, indicating that multi-directional thermal pathways could prevent excess heat from accumulating in a localized region and enable temperature distribution to be more uniform. For the latter, we quantify the thermal resistance from filler/filler interface by using a nonlinear model proposed by Foygel et al. to analyze the contribution of LM to heat dissipation (the detailed fitting and calculation process could be seen in Fig. S10). The interfacial thermal resistance was calculated as 9.1 × 10^–9^ m^2^ K W^−1^, which is one or two orders of magnitude less than that of previous polymer composites with three-dimensional BN network (Table S4), reflecting the bridge effect of LM facilitates heat transfer between BN sheets significantly [20, 65, 76, 77].

Apart from the capability of rapid heat conduction, enthalpy of melting ($\Delta$ *H*_mc_) and crystallization ($\Delta$ *H*_cp_) and its cycle durability are also indispensable parameters to evaluate the performance of PCM for battery thermal management, which enable undesirable thermal energy could be absorbed during phase transition. To control the battery to keep within a safe temperature range, we adjust the phase transition temperature under the alert temperature of normal operation (55 °C) by selecting PEG with average molecular weight of 4000 as matrix. DSC was performed to investigate the melting points and calculate the enthalpy of pure PEG and PEG/LM/BN composites through the endothermic and exothermic profiles (Fig. S11). The phase change composites (PCCs) with different loading of hybrid fillers all initiate the phase transition at the temperature lower than 50 °C, providing a thermal buffer against temperature propagation and avoiding thermal runaway risks. Moreover, although the incorporation of filler compromise with enthalpy of PCM, the PEG/LM/BN composite with the highest *k* still maintains 50.85% enthalpy of melting and 49.40% enthalpy of crystallization (absolute value =  ~ 80 J g^−1^). Additionally, the figure of merit (FOM), which is the product of *k*, density (*ρ*) and phase-change enthalpy ($\Delta$ *H*) (FOM = *k* × *ρ*×$\Delta$ *H*), was used as an integral parameter to conduct a more rational evaluation of a material’s ability to exchange thermal energy with its surroundings. Figure 3f and Table S5 show the comparison of FOM, and it could be found that our PEG/LM/BN composite presents much higher FOM and multifold *k* relative to BN-based PCCs reported in latest studies [54, 65–75]. Moreover, as shown in Fig. 3g, the enthalpy of melting and crystallization exhibits negligible reduction after 50 cyclic heating and cooling processes in which the endothermic and exothermic behavior as a function of time shows outstanding durability, which guarantee the PCM long-term and effective service for LIBs. We also investigated other safety performances that are similarly critical for the battery thermal management materials in practical application. Excellent shape stability, anticorrosion characteristic and flame resistance make our PCM more attractive for actual battery thermal management (Figs. S12–S14).

### Dual-Mode Battery Thermal Management of the PEG/LM/BN PCM

**Fig. 4:**
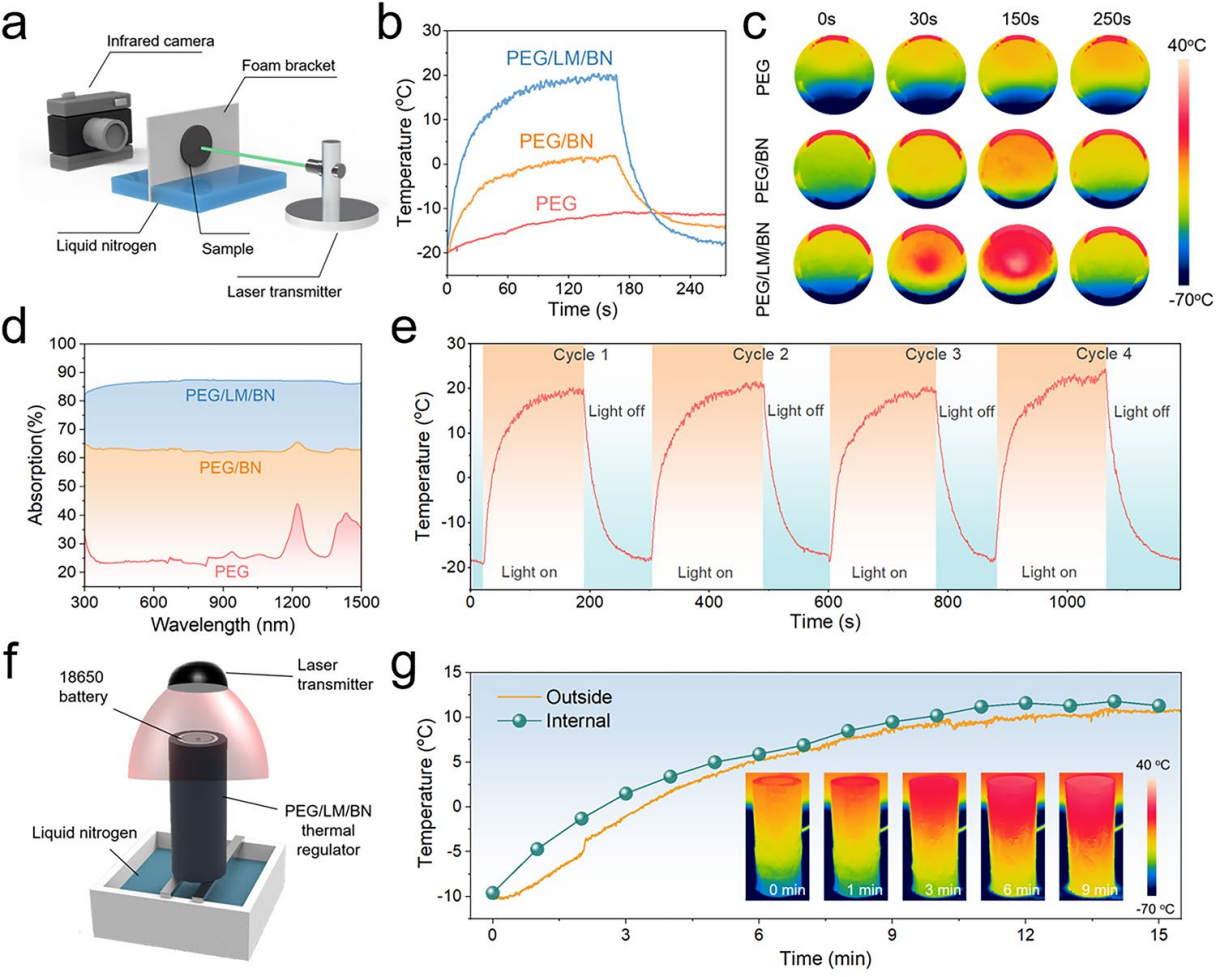
**a** Schematic of the test system used to investigate light-to-heat conversion capability including a laser light generating the powerful irradiation, an infrared (IR) camera recording the surface temperature of the sample and some liquid nitrogen placed in the foam chamber creating a low-temperature environment. **b** Average surface temperature of pure PEG, PEG/BN composite (loading content of 30 vol%) and PEG/LM/BN composite (loading content of 30 vol%) versus time of the test including light on (first half) and light off (the latter half). Note that the power of the laser light is 4 W. **c** Visualized light-to-heat conversion of pure PEG, PEG/BN composite (loading content of 30 vol%) and PEG/LM/BN composite (loading content of 30 vol%) recorded by IR camera. **d** UV–vis–NIR light absorption spectra of pure PEG, PEG/BN composite (loading content of 30 vol%) and PEG/LM/BN composite (loading content of 30 vol%). **e** Average surface temperature profiles of the PEG/LM/BN composite (loading content of 30 vol%) undergoing light on and light off after 4 cycles, showing the stability of heat generation at low temperature. **f** Schematic of heating the battery by utilizing the photothermal conversion ability of PEG/LM/BN thermal regulator. **g** Temperature profiles of the PEG/LM/BN thermal regulator upon the irradiation of the laser light

Excellent heat-conduction ability and phase-change durability are necessary for passive thermal management at high temperature. In view of the battery performance and safety at low temperature, it is essential for the PCM to possess the capability of heat generation to pre-heat the battery cell. Thanks to the design of bifunctional LM, our PEG/LM/BN composite could realize light-to-heat conversion even if it is placed where the temperature is lower than 0 °C. As a proof-of-concept experiment, we used a tailor-made foam chamber which not only fixed the PEG/LM/BN composite with the highest *k* but also held liquid nitrogen used to simulate a subzero environment, enabling the PEG/LM/BN composite to be over the liquid nitrogen. To evaluate the photothermal performance, the sample was irradiated by a laser (532 nm) and the temperature trend of the opposite surface was recorded by an infrared (IR) thermal camera to visualize the photothermal conversion process (Figs. 4a and S15). In Fig. S16, with laser power of 4 W, the average surface temperature was increased from − 20 to 15 °C (appropriate temperature range for battery operation) in one minute and the maximum surface temperature could reach nearly 30 °C. Also, note that the higher laser power, the more rapid heat generation and the higher surface temperature. For comparison, the pure PEG and PEG/BN composite with the same filler content were prepared as contrast samples and measured. However, as shown in Fig. 4b, c, the average surface temperatures of the pure PEG and PEG/BN composite are both below 0 °C with irradiation time increasing, which indicates the prominent contribution of LM to excellent light-to-heat conversion of PEG/LM/BN composites. This could be summarized as the uniform distribution of LM droplets within the composite and the plasmon resonance of LM, which enable broadband light absorption performance and high photothermal conversion efficiency (60.6%) of the composite (Figs. 4d and S17), contributing to the great photothermal performance. More importantly, our PEG/LM/BN composite exhibited splendid cycle durability in terms of light stimuli responsive heat generation, endowing the materials with the potential for low-temperature battery thermal management (Fig. 4e).

Based on the excellent properties of our PEG/LM/BN composites, we further tested the heating capability of the customized thermal regulator. In a sub-zero environment created by liquid nitrogen, our thermal regulator could pre-heat the cell once the laser transmitter was initiated (Fig. 4f). As shown in Fig. 4g, we monitored in real time the outside average temperature variation of the thermal regulator. By virtue of rapid photothermal conversion and uniform thermal transport, the heat flux was infused into the whole thermal regulator, resulting in the increasement of the outside surface temperature from − 10 to 10 °C. Given the intense thermal convection with low-temperature surroundings, we measured the temperature of the internal surface that closely clung to the cell by using a thermocouple. It could be found that the internal temperature was moderately higher than the outside temperature, proving the potential of the thermal regulator for working as a heater to enable the battery cell to operate at an appropriate temperature above freezing when faced with low-temperature challenge. Moreover, the cooperative work of our thermal regulator, advanced temperature sensor and laser transmitter makes it possible for a smart and self-adaptive battery thermal management system, enabling the LIBs could be pre-heated when the surrounding temperature becomes below 0 °C.

**Fig. 5:**
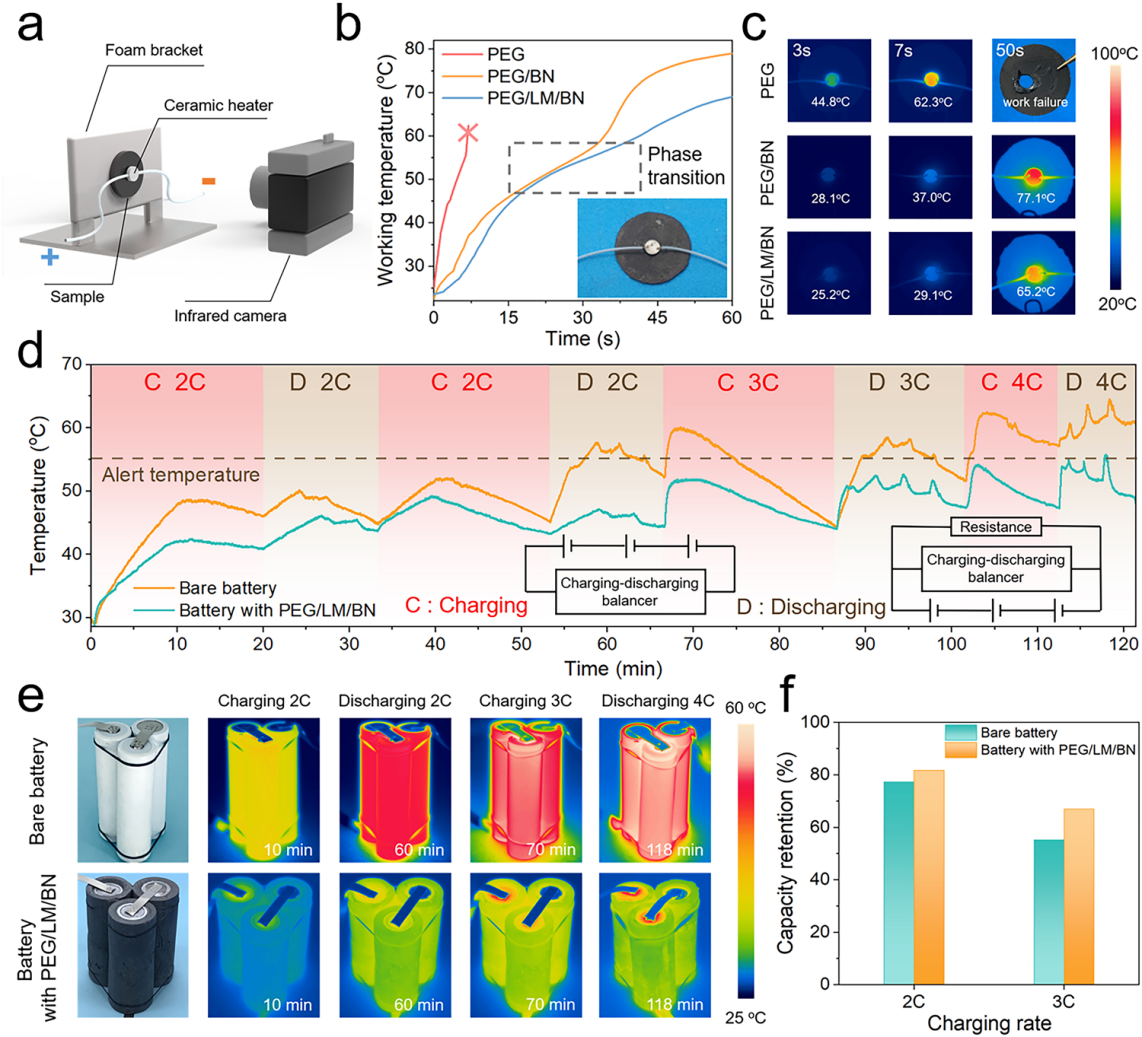
**a** Schematic of the test system used to investigate heat-dissipation performance including a powerful ceramic heater simulating localized overheating and an infrared (IR) camera recording the surface temperature of the sample. **b** Working temperature of the ceramic heater versus working time when thermally managed by pure PEG, PEG/BN composite (loading content of 30 vol%) and PEG/LM/BN composite (loading content of 30 vol%). The inset image is the digital photograph of a ceramic heater thermally managed by the PEG/LM/BN composite. **c** IR images at different heating times for thermal management of a point-like heat source by pure PEG, PEG/BN composite and PEG/LM/BN composite. **d** Battery temperature profiles for bare battery pack and battery pack with PEG/LM/BN thermal regulator under high-C charging and discharging. The inset shows the circuit connection. **e** Digital photographs of bare battery pack (top) and battery pack with PEG/LM/BN thermal regulator (bottom) and their IR images during the process of charging and discharging. **f** Capacity retention of bare battery and battery with PEG/LM/BN thermal regulator at different charging rate

In addition, to examine the high-temperature thermal management performance, a powerful ceramic heater with a diameter of 5 mm was attached to the center of the materials to simulate the localized overheating. We also recorded its temperature and took IR images after different working time through an IR camera (Fig. 5a). With a constant power of 0.7 W, in comparison with the pure PEG and PEG/BN composite, the temperature of the ceramic heater rose with the slowest rate when PEG/LM/BN composite was used as the heat spreader and its operation temperature behaved the lowest all the time, demonstrating the remarkable heat-dissipation capability of the PEG/LM/BN composite (Fig. 5b, c). Owing to the poor thermoconductive property of the pure PEG, the sample was destroyed by the enormous accumulated heat immediately. And when managed by PEG/BN or PEG/LM/BN composite, a gentle stage occurred during the process of heater’s temperature rise, which could be attributed to rapid heat transfer and thermal energy absorption caused by phase transition.

High temperature is derived from not only hot environment but also heat generated inside the LIBs during fast charging/discharging process. The charging/discharging rate, which is referred as C-rate, is defined as the charging or discharging current divided by the capacity of LIBs. To test the cooling performance of the PCM thermal regulator under high C-rate, we compared bare battery pack and battery pack with PEG/LM/BN thermal regulator during the fast charging and discharging cycles and the temperature profiles were recorded by IR camera (Figs. 5d, e and S18). The charging mode is constant current (CC). Here, we enabled three commercially available 18,650 LIB monomers (2300 mA h capacity) to be connected in series to construct a miniaturized battery pack. As shown in the IR images, the maximum temperature always occurs near the positive electrode. At a discharging of 2C, the maximum temperature of the bare battery pack could reach 57.7 °C which had gone beyond the alert temperature of 55 °C, whereas that of the battery pack with PEG/LM/BN thermal regulator was only 46.9 °C. With charging/discharging rate further increasing to 3C and even 4C, the bare battery pack demonstrated the maximum temperature of above 60 °C which seriously affected the batteries’ operation performance and possibly caused the risk of thermal runaway. However, the temperature of the battery pack with PEG/LM/BN thermal regulator was lowered by more than 10 °C compared with the bare battery pack, remaining below 55 °C, and showed lower fluctuations and more uniform distribution, preventing the overheating of the cells. Furthermore, we calculated the charging capacity retention to evaluate the influence of high temperature on the working performance (Fig. 5f). Although internal thermal condition induced by fast charging could contribute to battery aging and inevitable capacity loss [9], the battery pack with PEG/LM/BN thermal regulator afforded a charging capacity with 82% and 67% retention at 2C and 3C, which is 6.5% and 21.8% higher than the capacity delivered by the bare battery pack, respectively. Therefore, our thermal regulator can make contribution to saving energy, prolonging the working time of the LIBs and mitigating thermal safety issues.

## Conclusions

In summary, we reported a dual-mode battery thermal regulator to realize reliable operation of LIBs both in hot and cold environments on a single platform by an elaborate PEG/LM/BN PCM with hierarchical structure. The design of conformable configuration to the structure of LIBs in macro-scale and multidirectional thermal pathways in micro-scale enables rapid and uniform heat transfer (*k*_⊥_ = 8.8 W m^−1^ K^−1^ and *k*_//_ = 7.6 W m^−1^ K^−1^). The introduction of multifunctional LM not only reduces interfacial thermal resistance between the fillers by virtue of its high thermal conductivity and deformability but also serves as thermogenic component to achieve light-to-heat conversion to pre-heat the battery at low temperature. Other advantages include manufacturing adjustability, electric insulation, shape stability, anticorrosion characteristic and flame resistance. When applied in practical 18,650 LIBs system, our PCM thermal regulator could both act as a photo-responsive heater at sub-zero surroundings, and lower the battery’s temperature by more than 10 °C under high C-rate, affording 21.8% higher charging capacity. We hope such an adaptive battery thermal regulator could play a role in relieving the energy crisis and environmental pollution.

## Supplementary Information

Below is the link to the electronic supplementary material.

Supplementary file1 (PDF 2273 kb)
