# Supplementary material for: Bifunctional Liquid Metals Allow Electrical Insulating Phase Change Materials to Dual-Mode Thermal Manage the Li-Ion Batteries
Source: Nanomicro Lett. 2022 Oct 10;14:202. doi: 10.1007/s40820-022-00947-w (PMC9551009; doi:10.1007/s40820-022-00947-w)
Supplement: Supplementary file 1 — Supplementary file1 (PDF 2273 kb) [file 40820_2022_947_MOESM1_ESM.pdf]

Supporting Information for

Bifunctional Liquid Metals Allow Electrical Insulating Phase Change Materials to Dual-Mode Thermal Manage the Li-Ion Batteries

Cong Guo[1, #], Lu He[1, #], Yihang Yao[1], Weizhi Lin[1], Yongzheng Zhang[1, 2], Qin Zhang[1], Kai Wu[1, *], Qiang Fu[1, *]

[1]College of Polymer Science and Engineering, State Key Laboratory of Polymer Materials Engineering, Sichuan University, Chengdu 610065, P. R. China

[2]Department of Polymer Science and Engineering, School of Chemical Engineering, Nanjing University of Science and Technology, Nanjing 210094, P. R. China

[#]Cong Guo and Lu He contributed equally to this work.

*Corresponding authors. E-mail: kaiwu@scu.edu.cn (K. Wu), qiangfu@scu.edu.cn (Q. Fu)

Supplementary Tables and Figures

Table S1 Nomenclature

k	Thermal conductivity
$k_{\perp}$	Through-plane thermal conductivity
$k_{//}$	In-plane thermal conductivity
vol %	Volume fraction
C	Rate of charge/discharge of battery
Ah	Ampere hour
V	Volt
^{o}C	Degree Celsius
s	Second
ρ	Density
C_{p}	Specific heat
ΔH_{mc}	enthalpy of melting
ΔH_{cp}	enthalpy of crystallization
W	Watts
mm	Millimeter
μm	Micrometer
K	Kelvin

Table S2 Abbreviations used in this work

Abbreviation	Description
PCM	Phase change material
LIBs	Lithium-ion batteries
LM	Liquid metal
BN	Boron nitride
PEG	Polyethylene glycol
SCMC	Sodium carboxymethylcellulose
PTFE	polytetrafluoroethylene
SEM	Scanning electron microscopy
AFM	Atomic force microscopy
XPS	X-ray photoelectron spectroscopy
DSC	Differential scanning calorimetry
DFT	Density functional theory

FES	Finite element simulation
PCC	Phase change composite
FOM	Figure of merit
IR	Infrared

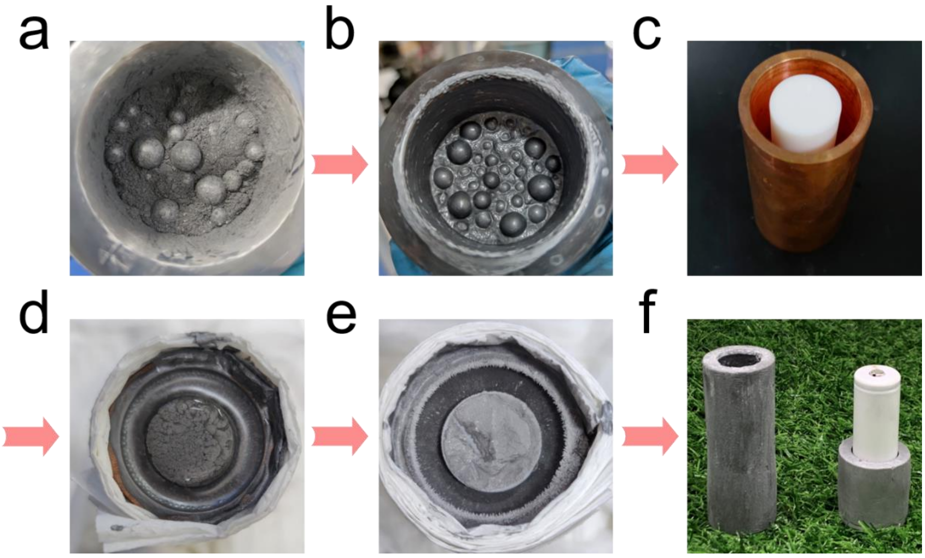

Fig. S1 Actual photos showing the fabrication process of LM/BN skeleton. **a** LM/BN binary hybrid fillers fabricated by ball milling. **b** The LM/BN suspension that is consist of LM/BN hybrids and 1 wt% SCMC deionized water solution fabricated by ball milling. **c** Structured copper mold with adiabatic Teflon stick in the center. **d** The LM/BN suspension poured into a customized mold. **e** The frozen slurries after ice-template growth. **f** The resultant PEG/LM/BN PCM thermal regulator which is conformable to the structure of a 18650 LIB

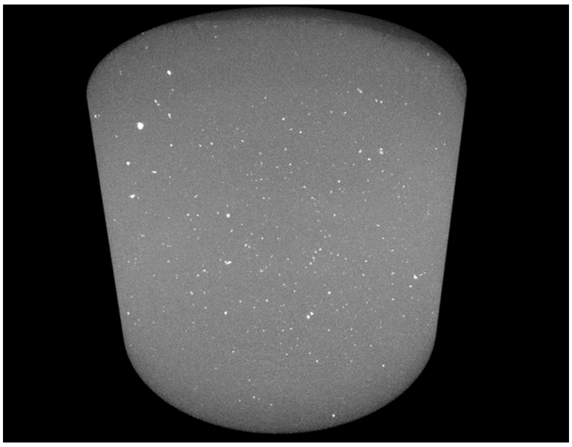

Fig. S2 Micro computed tomography image of the PEG/LM/BN composite

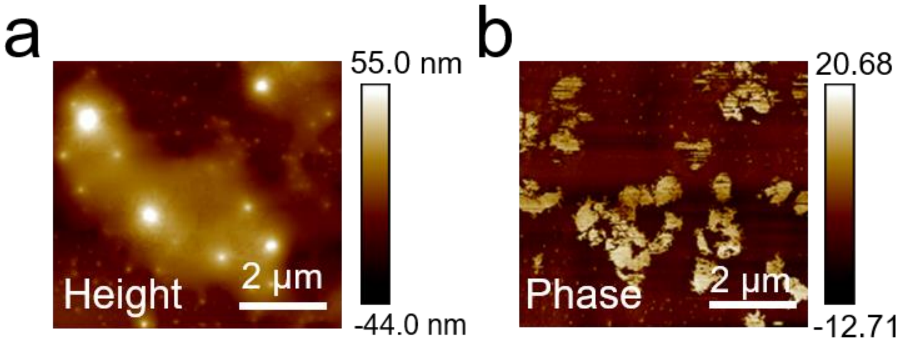

Fig. S3 Atomic force microscopy (AFM) images of LM/BN binary hybrid filler. **a** is the height image and the **b** is the phase image

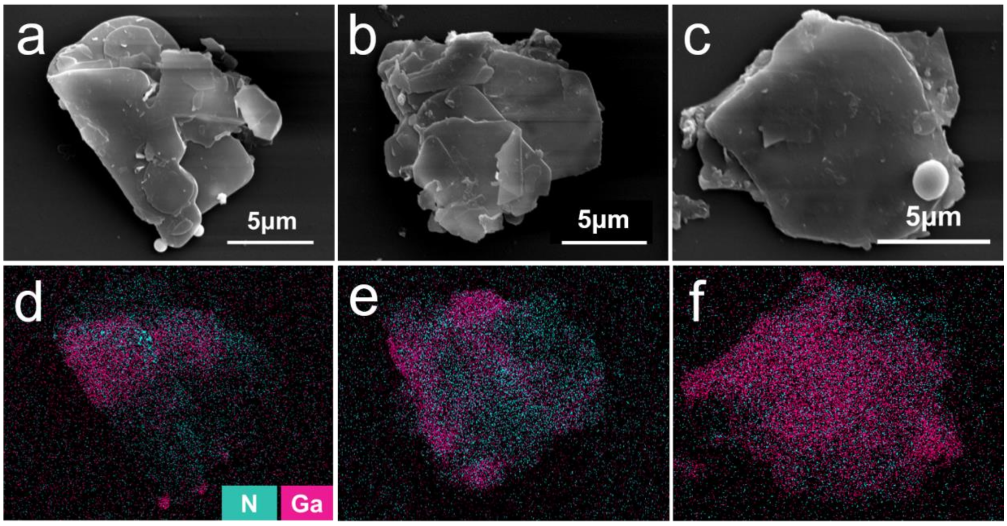

Fig. S4 Scanning electron microscopy (SEM) images of LM/BN binary hybrid fillers of which lateral sizes are about 10 μm with LM content of **a** 5 vol%, **b** 10 vol% and **c** 50 vol%, and **d-f** the corresponding elemental mapping images

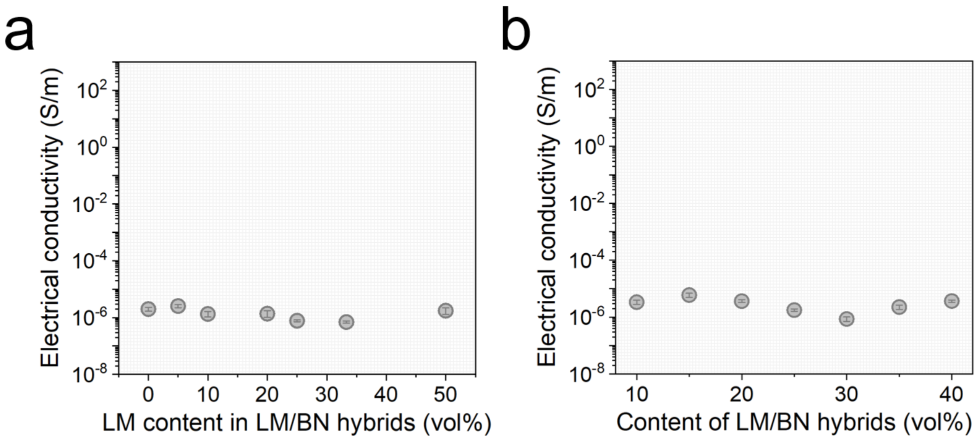

Fig. S5 a Electrical conductivity of the PEG/LM/BN composites as a function of the LM content in hybrid fillers (~10 μm). Note that the total volume fraction of LM/BN hybrid fillers in composites is a constant value of 30%. **b** Electrical conductivity of the PEG/LM/BN composites as a function of the volume fraction of the binary LM/BN hybrid fillers (~18 μm). Note that the LM content in LM/BN hybrid fillers is 10 vol%

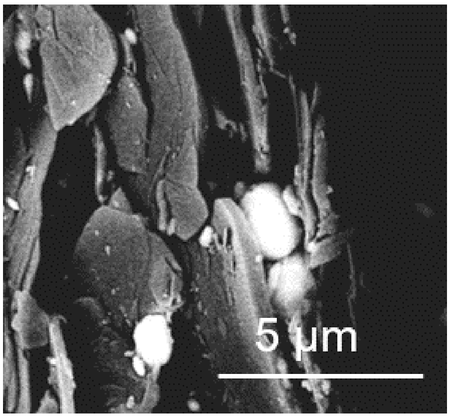

Fig. S6 Backscatter electron image taken from the longitudinal section of the PEG/LM/BN composites incorporating 30 vol% LM/BN hybrid fillers (~10 μm) with LM content of 10 vol %. Note that the brighter region represents higher ordinal element. Hence, the white regions are LM. The grey regions are BN sheets

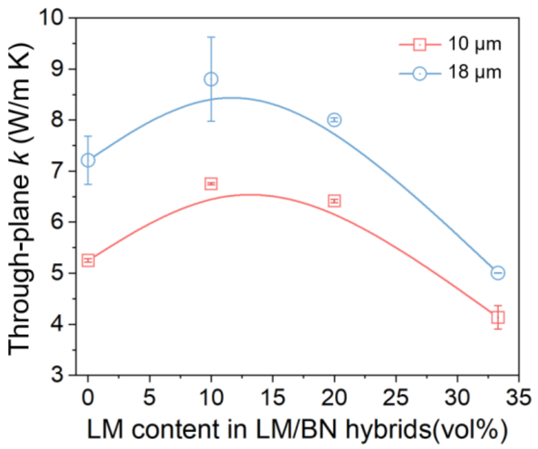

Fig. S7 Through-plane thermal conductivities (*k*) of the PEG/LM/BN composites as a function of the LM content in hybrid fillers with lateral size of ~ 10 and 18 μm. Note that the total volume fraction of LM/BN hybrid fillers in composites is a constant value of 30%

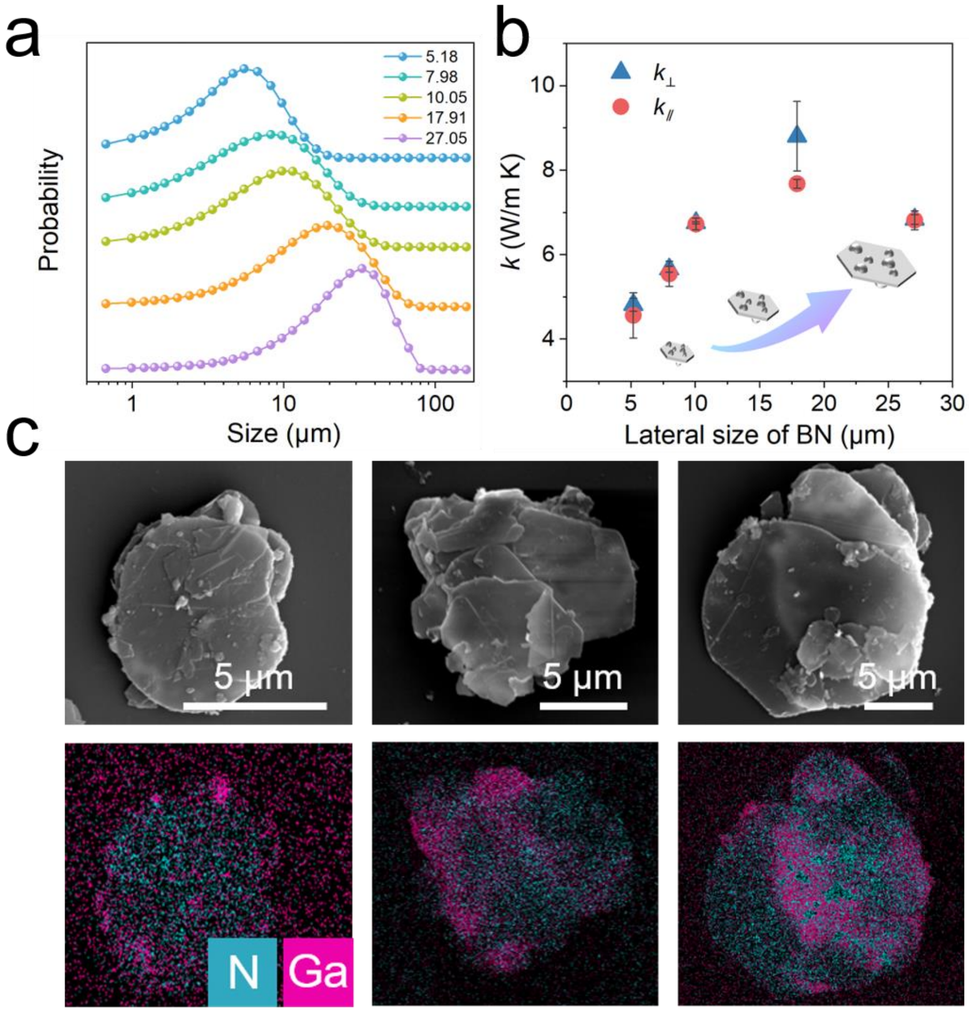

Fig. S8 a Statistic of lateral size of LM/BN hybrids. **b** Through-plane *k* ($k_{\perp}$) and in-plane *k* ($k_{//}$) of the PEG/LM/BN composites as a function of the lateral size of hybrid fillers with the same LM modification content (10 vol%). Note that the hybrid filler volume fractions of the fabricated composites were maintained at 30 vol% **c** Scanning electron microscopy images of LM/BN binary hybrid fillers with lateral sizes of about 5, 10 and 18 μm and LM content of 10 vol% and the corresponding elemental mapping images

Table S3 Comparison of $k_{\perp}$ and $k_{//}$ of our PEG/LM/BN composites with previously reported BN-based polymer composites

**Materials**	$k_{//}$ **(W/m K)**	$k_{\perp}$ **(W/m K)**	**Refs.**
This work			
PEG/LM/BN	7.68	8.8	
BN-based polymer composites reported in the literature			
PDMS/BNNS	0.59	1.54	[S1]
CER/BN@Fe	0.99	0.98	[S2]
Epoxy/SiCNW	1.45	1.67	[S3]
CNF/BNNT	21.39	1.68	[S4]
Epoxy/BNNS	6.54	0.70	[S5]
Epoxy/SiC+BNNS	1.43	4.22	[S6]
PEG/BNNS	4.76	1.29	[S7]
Epoxy/BNNS	2.40	2.85	[S8]
CNF/BNNS	12.79	0.13	[S9]
HDPE/BN	3.57	0.62	[S10]
HDPE/BN	1.60	1.60	[S10]
PVDF/BNNS	10.40	0.49	[S11]
PVDF/BNNS	16.30	0.78	[S11]
Epoxy/BNNS	0.40	0.40	[S12]
PDMS/BN	11.05	1.15	[S13]
TPU/BN	2.56	0.9	[S14]
PI/BN	2.81	0.73	[S15]
PVA/BN	3.92	0.44	[S16]
PVA/BN	8.28	0.63	[S17]
PEG/BN	4.41	2.55	[S18]
PC/BN	3.09	0.86	[S19]
CNF/BNNS	22.67	1.08	[S20]
CNF/BNNS	14.95	0.35	[S20]
Epoxy/BN	0.70	0.70	[S21]
Epoxy/BN	1.20	1.20	[S22]

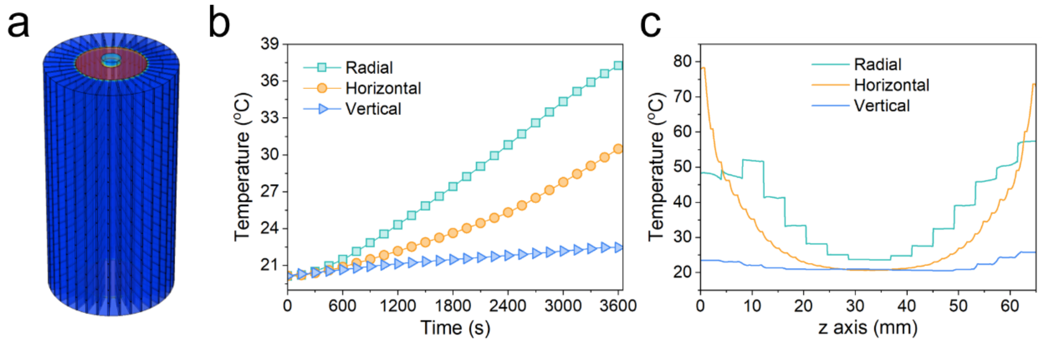

Fig. S9 a Initial temperature distribution of simulated model of PEG/LM/BN composites with radial orientations used as battery thermal regulator. **b** Outside surface average temperature of PEG/LM/BN composites with various orientation structure as a function of heating time. **c** Outside surface temperature distribution of PEG/LM/BN composites with various orientation structure along the z axis as the increase of height at 3600 s

We simulated the PEG/LM/BN composites thermal regulators with radial, horizontal and vertical thermal pathways to analyze the impact of structure design on the performance of battery thermal management. To investigate the uniformity of heat transfer, the regions of the electrodes were set as the main heat source (80 °C). During the heat dispassion process, the

temperature and distribution of heat flux vector within composite are simulated based on classical Flourier Law:

$$q = - k \times \frac{\Delta T}{\Delta x} \tag{S1}$$

where q is the heat flux, k is the thermal conductivity, and $\frac{\Delta T}{\Delta x}$ is the temperature gradient, respectively.

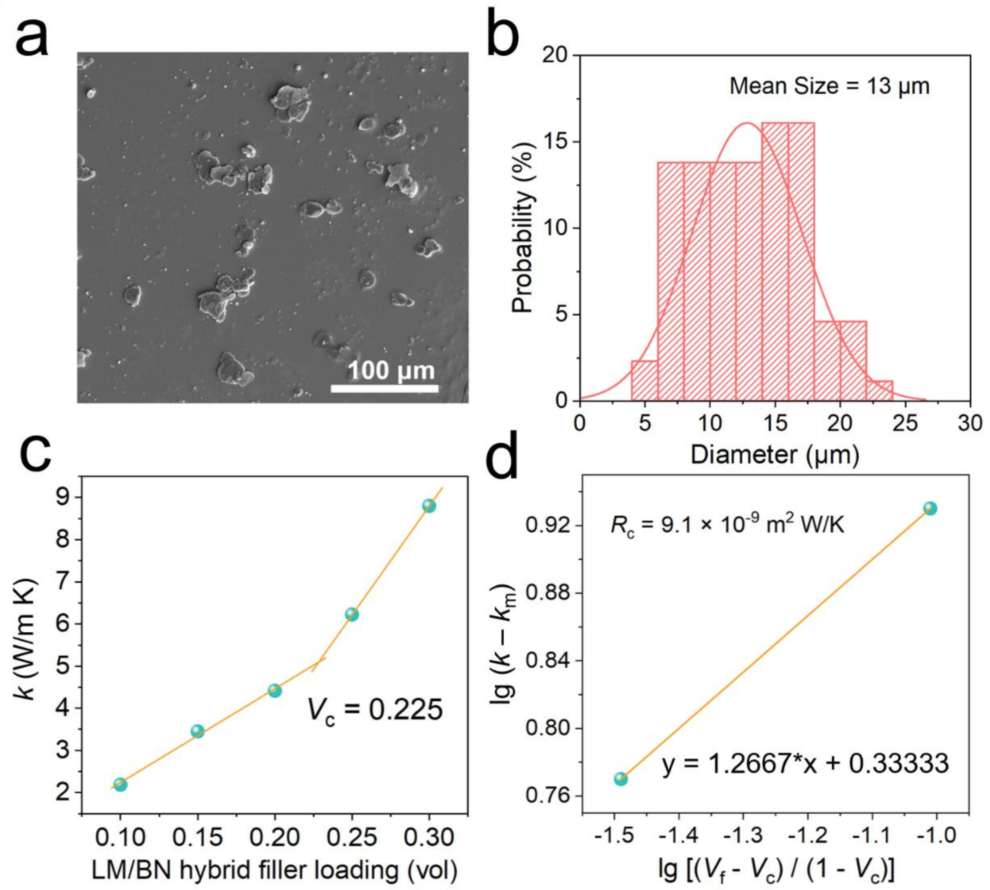

Fig. S10 a SEM image of LM/BN binary hybrid filler and **b** the statistic of its lateral size. **c** The tangent process of experimental data for PEG/LM/BN composites to obtain Vc. **d** The fitting process of Foygel model for PEG/LM/BN composites

The Foygel's nonlinear mode is used to calculate the contact thermal resistance between fillers. The equations are listed as follows:

$$k - k_m = k_0 (V_f - V_c / 1 - V_c)^{\tau} \tag{S2}$$

$$R_c = S / k_0 L (V_c)^{\tau} \tag{S3}$$

Where k is the thermal conductivity of PEG/LM/BN composites, V_f is the volume fraction of fillers, V_c is the critical volume fraction of fillers. L (13 μm) is the LM/BN size, obtained from Fig. S10a, b. k_0 is a preexponential factor ratio and τ is a conductivity exponent, which are both related to BN platelet contribution. In Fig. S10c, we did the tangent for the original experimental data of PEG/LM/BN composites and got the value of V_c (0.225). The equation $k - k_m = k_0 (V_f - V_c / 1 - V_c)^{\tau}$ was then transformed into a linear form: $\lg (k - k_m) = \lg k_0 + \tau \times \lg [(V_f - V_c) / (1 - V_c)]$. After fitted, as shown in Fig. S10d, k_0 and τ were obtained ($k_0 = 19.93$, $\tau = 0.32$). S is the average contact area between adjacent two BN platelet. We assume that 1/100 of each LM/BN surface take part in the heat conduction of the skeleton. Therefore, the resultant value of interfacial thermal resistance was calculated as 9.1×10^{-9} m^2 K W^{-1}.

Table S4 Comparison of thermal resistance from filler/filler interface of our PEG/LM/BN composite with that of previously reported polymer composites with three-dimensional BN network

Materials	R_c (m^2 K W^{-1})	Refs.
Epoxy/3D-BN	5.6×10^{-7}	[S23]
Epoxy/BN-GNP	8.4×10^{-8}	[S24]
Epoxy/BN-rGO	4.5×10^{-8}	[S22]
Epoxy/radial BN-GO	2.1×10^{-8}	[S18]
PEG/LM/BN	9.1×10^{-9}	This work

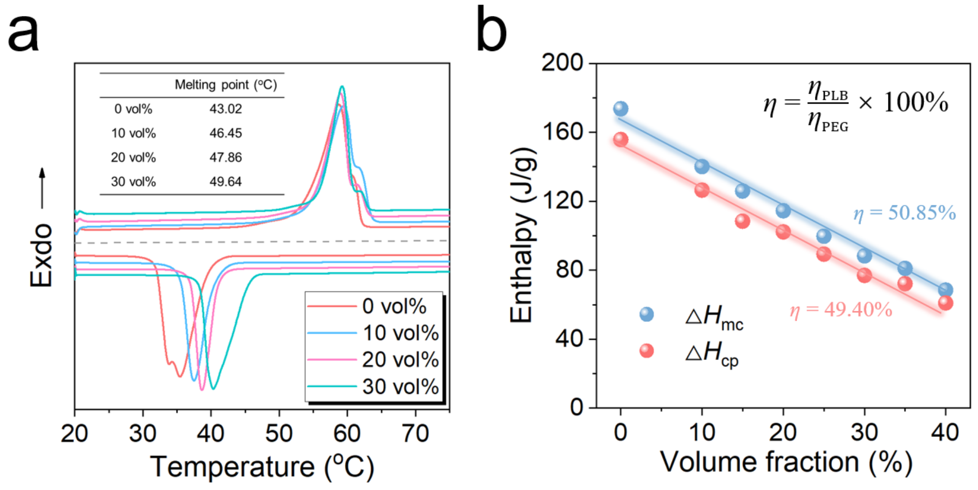

Fig. S11 a Differential scanning calorimetry (DSC) curves of pure PEG and PEG/LM/BN composites with different contents of LM/BN hybrid fillers (lateral size of ~18μm, LM in LM/BN hybrid fillers of 10 vol%). **b** Calculated enthalpy of melting and crystallization of the PEG/LM/BN composites with different contents of LM/BN hybrid fillers

Table S5 Comparison of the figure of merits and *k* of BN-based phase change composites from the latest studies

PCM	Filler	Content	ΔH (J/g)	*k* (W/m K)	*ρ* (g/cm^3)	FOM	Refs.
PEG	BNNS	10vol%	136.80	4.76	1.25	812.04	[S7]
PEG	BN-GNP	31 vol%	116.80	1.33	1.42	219.94	[S24]
PEG	BN	23 wt%	138.20	2.36	1.28	417.15	[S25]
PEG	GO/BN	28.7 wt %	143.60	3.18	1.33	606.07	[S26]
PEG	BN	16.3vol%	134.40	3.26	1.32	577.95	[S27]
PEG	GO/BN	0.78/6.76wt%	160.70	0.79	2.31	293.20	[S28]
PW	BN	18wt%	165.40	0.85	0.99	139.29	[S29]
PW	h-BN	10 wt%	177.00	0.53	0.92	86.76	[S30]
PW	BN	20wt%	147.70	0.44	1.01	65.52	[S31]
PW	BNNS	40 wt%	80.15	3.47	1.21	335.54	[S32]
SAT	h-BN	5wt%	210.00	1.20	1.56	392.59	[S33]
C18 and SA	h-BN	10 wt%	207.60	0.32	0.88	58.78	[S34]
PEG	LM/BN	30vol%	80.00	8.80	1.65	1162.66	This work

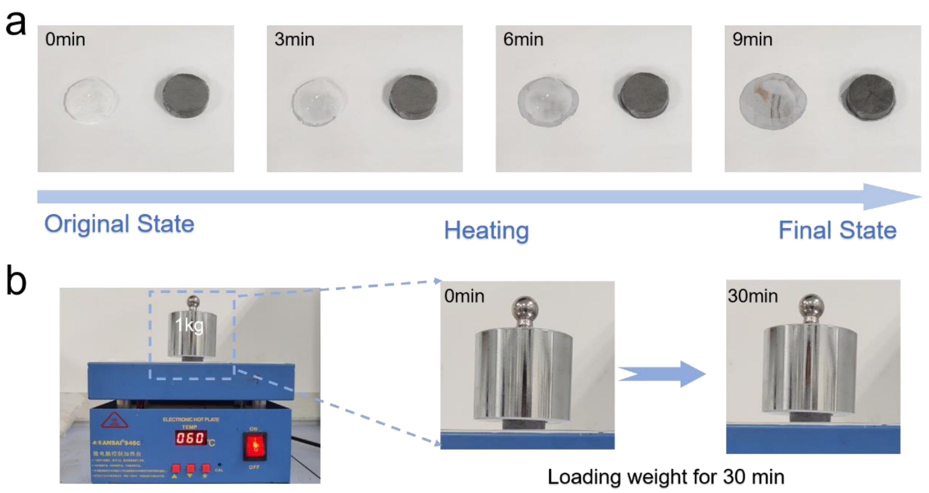

Fig. S12 Shape stability. **a** Optical photographs of PEG (left) and PEG/LM/BN composite (right) on a 60 °C hot plate. **b** Pressure resistance of a PEG/LM/BN composites at a hot stage of 60 °C showing excellent shape stability

It can be observed from Fig. S12a, PEG lost its shape quickly and spread out on the heating stage during the heating process. However, the PEG/LM/BN composite retained its shape at 60 °C, which indicates that the LM/BN skeleton effectively improved the shape stability of the composites owing to the existance of the capillary and surface tension force between PEG and the LM/BN skeleton. In Fig. S12b, a 1kg weight was put on the PEG/LM/BN composites at 60 °C for 30 min. There was no obvious leakage of molten PEG, which further suggests its excellent leakage-proof characteristic, paving the way for our composites' practical applicatipn in passive battery thermal management.

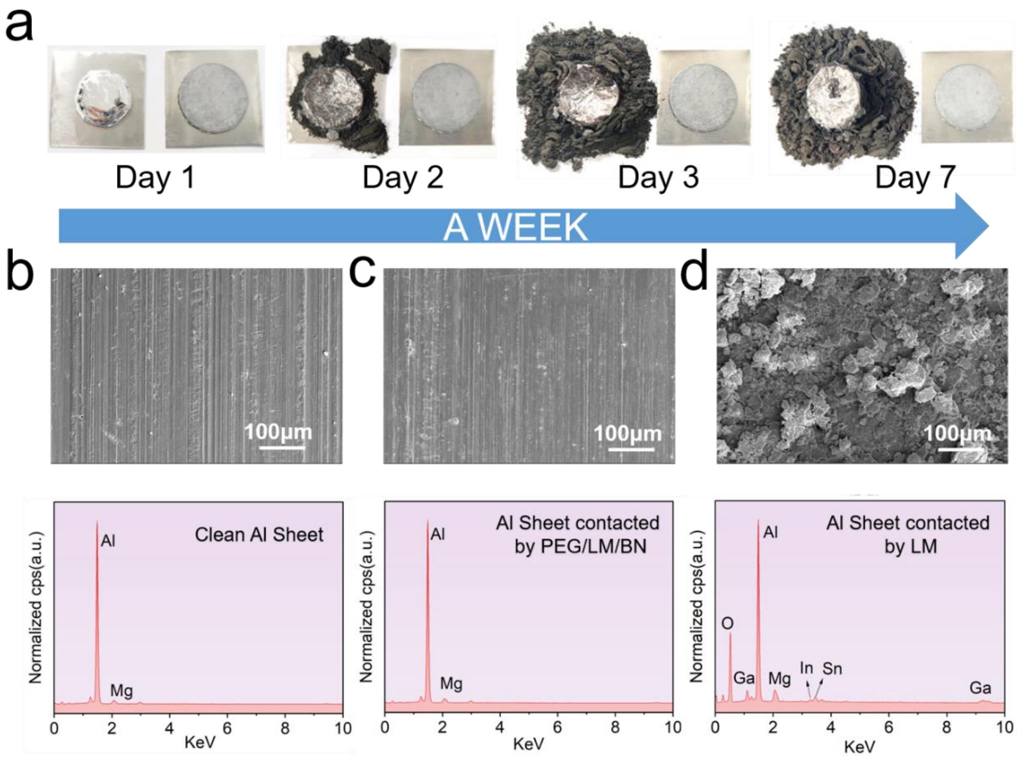

Fig. S13 Anticorrosion characteristic. **a** Optical photographs of corrosion of aluminum surface after contacting with LM (left) and our PEG/LM/BN composite (right). SEM images and energy-dispersive spectra (EDS) results of **b** clean aluminum sheet, **c** aluminum surface contacted by the PEG/LM/BN composite for a week and **d** aluminum surface contacted by liquid metal for a week

As shown in Fig. S13, LM and PEG/LM/BN composites were put on the clean aluminum sheets for a week to investigate the anticorrosion performance. The aluminum contacted by LM was corroded quickly and totally, while the aluminum contacted by the PEG/LM/BN composite maintained clean as its original state. And the aluminum sheet coated with LM became rough and powdery. The increasement of gallium and oxygen in the EDS images show the corrosion characteristic of liquid metals. However, the aluminum sheet after contacting with the PEG/LM/BN composite did not have any traces of corrosion and superfluous elements on the EDS images, demonstrating no reaction between aluminum and PEG/LM/BN composites.

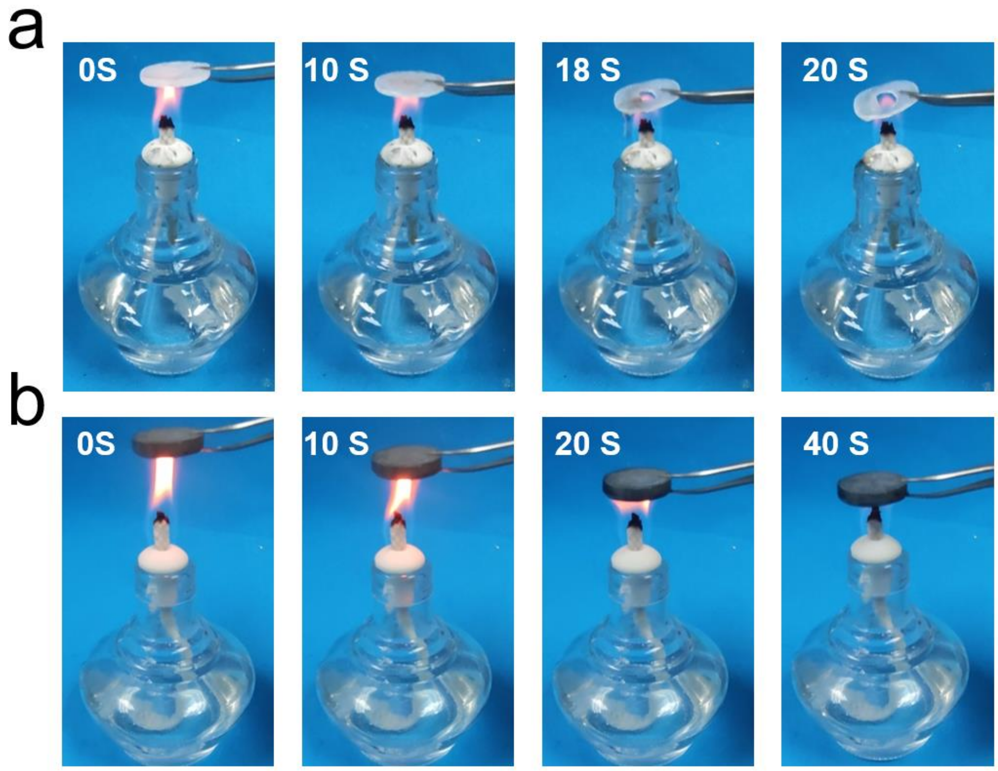

Fig. S14 Flame resistance. Optical photographs of **a** PEG and **b** PEG/LM/BN composites after contacting the flame, showing the excellent flame resistance of PEG/LM/BN composites

Obviously, the PEG materials melt and decompose at high temperature, thus leading to rapid flame spread and continuous burning until the sample is completely combusted (Fig. S14a). However, as shown in Fig. S14b, the structural integrity of the PEG/LM/BN composite could be well kept and the flame showed rapid self-extinguishing phenomenon, indicating its excellent flame resistance.

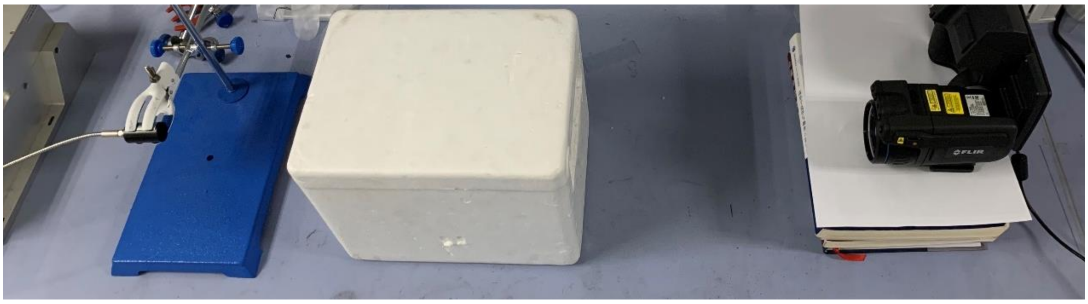

Fig. S15 The digital photo of the test system used to investigate light-to-heat conversion capability including a laser light generating the powerful irradiation (left), an infrared (IR) camera recording the surface temperature of the sample (right) and some liquid nitrogen placed in the foam chamber creating a low-temperature environment (middle)

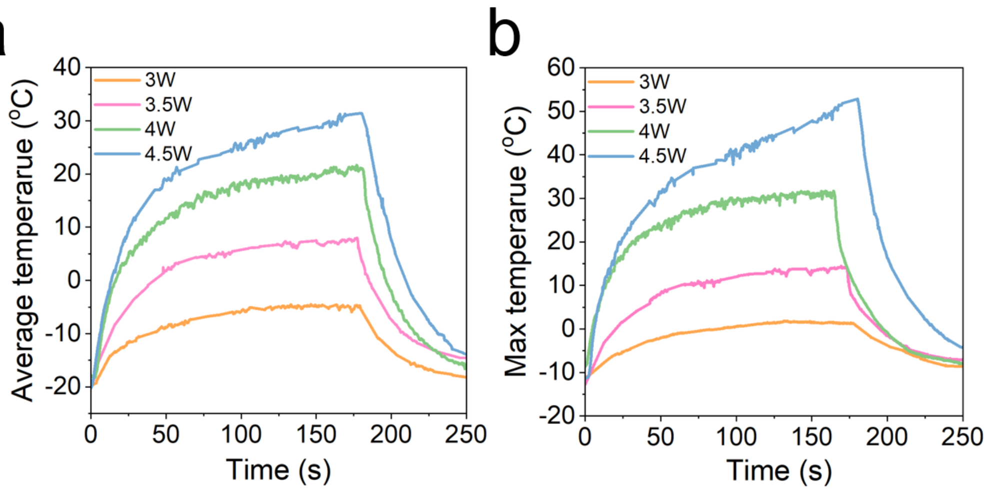

Fig. S16 a Average surface temperature and **b** maximum surface temperature of PEG/LM/BN composite (loading content of 30 vol %) irradiated by laser light of different power versus time of the test including light on (first half) and light off (the latter half)

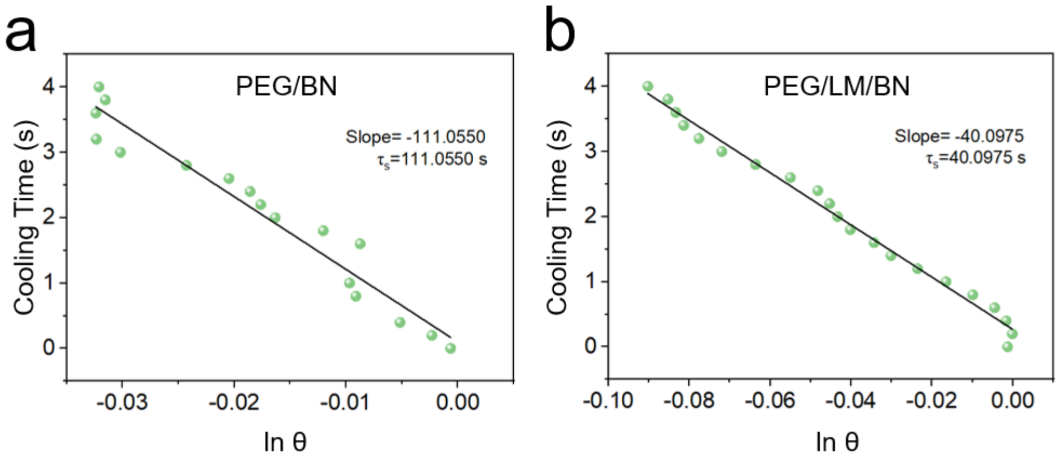

Fig. S17 Corresponding cooling time versus ln θ linear curve for calculation of photothermal conversion efficiency of **a** PEG/BN and **b** PEG/LM/BN

The photothermal conversion efficiency was calculated as follows:

Based on the balance of system energy:

$$\sum_i m_i C_{pi} \frac{dT}{dt} = Q_s - Q_{loss} \quad (S4)$$

Where m_i (PEG/BN composite (4.116 g/0.337 g); PEG/LM/BN composite (0.4116 g/0.0947 g/0.303 g)) and C_{pi} (PEG: 1.79 J/(g K); LM: 0.4 J/(g K); BN: 0.9 J/(g K)) are the mass and heat capacity of the composite. Q_s is the photothermal heat energy input from the laser light and Q_{loss} is the thermal energy lost to the surroundings.

When the temperature of the samples is maximum, the system is in balance:

$$Q_s = Q_{loss} = hS\Delta T_{max} \quad (S5)$$

Where h means heat transfer coefficient, S is the surface area of the sample being irradiated and ΔT_{max} means the maximum temperature change.

Subsequently, the photothermal conversion efficiency:

$$\eta = \frac{hS\Delta T_{max}}{I(1-10^{-A_{sos}})} \quad (S6)$$

where I is the laser power (4 W) and A_{sos} is the absorbance of the composites (PEG/BN composite: 0.553; PEG/LM/BN composite: 0.865) at the wavelength of 532 nm. And the hS can obtain as follows:

It is defined:

$$\theta = \frac{T-T_s}{T_m-T_s};\ \tau_s = \frac{\sum_i m_i C_{pi}}{hS} \quad (S7)$$

where T means the temperature of the samples, T_s means the temperature of the surroundings (-60 ^{o}C), T_m means the maximum temperature and τ_s is the system time constant.

So that $d\theta/dt = Q_s/(\tau_s hS\Delta T_m) - \theta/\tau_s$

when $Q_s = 0$ (turn off the laser light), therefore $d\theta/dt = -\theta/\tau_s$, and $t = -\tau_s \ln\theta$;

so hS could be calculated.

The τ_s of PEG/BN composite is 111.055 s and the τ_s of PEG/LM/BN composite is 40.0975 s. The photothermal conversion efficiency η of PEG/BN composite is 20.1% and the photothermal conversion efficiency η of PEG/LM/BN composite is 60.6%.

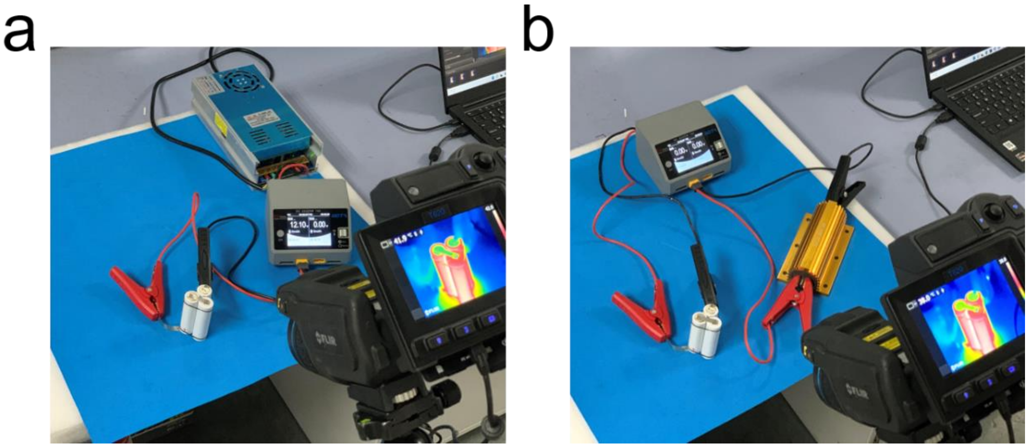

Fig. S18 Digital photos of device design showing the circuit connection for **a** charging and **b** discharging process by using a charge-discharge balancer and a large-power electric resistance

Supplementary References

[S1] J. Chen, X. Huang, B. Sun, Y. Wang, Y. Zhu et al., Vertically aligned and interconnected boron nitride nanosheets for advanced flexible nanocomposite thermal interface materials. ACS Appl. Mater. Interfaces **9**(36), 30909-30917 (2017). https://doi.org/10.1021/acsami.7b08061

[S2] Z. Su, H. Wang, J. He, Y. Guo, Q. Qu et al., Fabrication of thermal conductivity enhanced polymer composites by constructing an oriented three-dimensional staggered interconnected network of boron nitride platelets and carbon nanotubes. ACS Appl. Mater. Interfaces **10**(42), 36342-36351 (2018). https://doi.org/10.1021/acsami.8b09703

[S3] Y. Yao, X. Zhu, X. Zeng, R. Sun, J.B. Xu et al., Vertically aligned and interconnected SiC nanowire networks leading to significantly enhanced thermal conductivity of polymer composites. ACS Appl. Mater. Interfaces **10**(11), 9669-9678 (2018). https://doi.org/10.1021/acsami.8b00328

[S4] X. Zeng, J. Sun, Y. Yao, R. Sun, J.B. Xu et al., A combination of boron nitride nanotubes and cellulose nanofibers for the preparation of a nanocomposite with high thermal conductivity. ACS Nano **11**(5), 5167-5178 (2017). https://doi.org/10.1021/acsnano.7b02359

[S5] J. Han, G. Du, W. Gao, H. Bai, An anisotropically high thermal conductive boron nitride/epoxy composite based on nacre-mimetic 3D network. Adv. Funct. Mater.

29(13), 1900412 (2019). https://doi.org/10.1002/adfm.201900412

[S6] C. Xiao, Y. Guo, Y. Tang, J. Ding, X. Zhang et al., Epoxy composite with significantly improved thermal conductivity by constructing a vertically aligned three-dimensional network of silicon carbide nanowires/boron nitride nanosheets. Compos. B Eng. **187**, 107855 (2020). https://doi.org/10.1016/j.compositesb.2020.107855

[S7] C. Lei, K. Wu, L. Wu, W. Liu, R. Du et al., Phase change material with anisotropically high thermal conductivity and excellent shape stability due to its robust cellulose/BNNSs skeleton. J. Mater. Chem. A **7**(33), 19364-19373 (2019). https://doi.org/10.1039/C9TA05067A

[S8] X. Zeng, Y. Yao, Z. Gong, F. Wang, R. Sun et al., Ice-templated assembly strategy to construct 3D boron nitride nanosheet networks in polymer composites for thermal conductivity improvement. Small **11**(46), 6205-6213 (2015). https://doi.org/10.1002/smll.201502173

[S9] K. Wu, J. Fang, J. Ma, R. Huang, S. Chai et al., Achieving a collapsible, strong, and highly thermally conductive film based on oriented functionalized boron nitride nanosheets and cellulose nanofiber. ACS Appl. Mater. Interfaces **9**(35), 30035-30045 (2017). https://doi.org/10.1021/acsami.7b08214

[S10] X. Zhang, J. Zhang, L. Xia, C. Li, J. Wang et al., Simple and consecutive melt extrusion method to fabricate thermally conductive composites with highly oriented boron nitrides. ACS Appl. Mater. Interfaces **9**(27), 22977-22984 (2017). https://doi.org/10.1021/acsami.7b05866

[S11] J. Chen, X. Huang, B. Sun, P. Jiang, Highly thermally conductive yet electrically insulating polymer/boron nitride nanosheets nanocomposite films for improved thermal management capability. ACS Nano **13**(1), 337-345 (2018). https://doi.org/10.1021/acsnano.8b06290

[S12] J. Chen, X. Huang, Y. Zhu, P. Jiang, Cellulose nanofiber supported 3D interconnected BN nanosheets for epoxy nanocomposites with ultrahigh thermal management capability. Adv. Funct. Mater. **27**(5), 1604754 (2017). https://doi.org/10.1002/adfm.201604754

[S13] H. Hong, Y.H. Jung, J.S. Lee, C. Jeong, J.U. Kim et al., Anisotropic thermal conductive composite by the guided assembly of boron nitride nanosheets for flexible and stretchable electronics. Adv. Funct. Mater. **29**(37), 1902575 (2019). https://doi.org/10.1002/adfm.201902575

[S14] J. Liu, W. Li, Y. Guo, H. Zhang, Z. Zhang, Improved thermal conductivity of thermoplastic polyurethane via aligned boron nitride platelets assisted by 3D printing. Compos. Part A Appl. Sci. Manuf. **120**, 140-146 (2019). https://doi.org/10.1016/j.compositesa.2019.02.026

[S15] H. Wang, D. Ding, Q. Liu, Y. Chen, Q. Zhang, Highly anisotropic thermally conductive polyimide composites via the alignment of boron nitride platelets. Compos. B Eng. **158**, 311-318 (2019). https://doi.org/10.1016/j.compositesb.2018.09.104

[S16] B.H. Xie, X. Huang, G.J. Zhang, High thermal conductive polyvinyl alcohol composites with hexagonal boron nitride microplatelets as fillers. Compos. Sci. Technol. **85**, 98-103 (2013). https://doi.org/10.1016/j.compscitech.2013.06.010

[S17] J. Zhang, X. Wang, C. Yu, Q. Li, Z. Li et al., A facile method to prepare flexible boron nitride/poly(vinyl alcohol) composites with enhanced thermal conductivity. Compos. Sci. Technol. **149**, 41-47 (2017). https://doi.org/10.1016/j.compscitech.2017.06.008

[S18] D. Liu, C. Lei, K. Wu, Q. Fu, A multidirectionally thermoconductive phase change material enables high and durable electricity via real-environment solar-thermal-electric conversion. ACS Nano **14**(11), 15738-15747 (2020). https://doi.org/10.1021/acsnano.0c06680

[S19] N. Sun, J. Sun, X. Zeng, P. Chen, J. Qian et al., Hot-pressing induced orientation of boron nitride in polycarbonate composites with enhanced thermal conductivity. Compos. Part A Appl. Sci. Manuf. **110**, 45-52 (2018). https://doi.org/10.1016/j.compositesa.2018.04.010

[S20] Z. Hu, S. Wang, G. Chen, K. Wu, J. Shi et al., An aqueous-only, green route to exfoliate boron nitride for preparation of high thermal conductive boron nitride nanosheet/cellulose nanofiber flexible film. Compos. Sci. Technol. **168**, 287-295 (2018). https://doi.org/10.1016/j.compscitech.2018.09.020

[S21] J. Ma, N. Luo, Z. Xie, F. Chen, Q. Fu, Preparation of modified hexagonal boron nitride by ball-milling and enhanced thermal conductivity of epoxy resin. Mater. Res. Exp. **6**(10), 1050d8 (2019). https://doi.org/10.1088/2053-1591/ab432a

[S22] Y. Yao, J. Sun, X. Zeng, R. Sun, J.B. Xu et al., Construction of 3D skeleton for polymer composites achieving a high thermal conductivity. Small **14**(13), 1704044 (2018). https://doi.org/10.1002/smll.201704044

[S23] J. Hu, Y. Huang, Y. Yao, G. Pan, J. Sun et al., Polymer composite with improved thermal conductivity by constructing a hierarchically ordered three-dimensional interconnected network of BN. ACS Appl. Mater. Interfaces **9**(15), 13544-13553 (2017). https://doi.org/10.1021/acsami.7b02410

[S24] J. Yang, L.S. Tang, R.Y. Bao, L. Bai, Z.Y. Liu et al., Largely enhanced thermal conductivity of poly (ethylene glycol)/boron nitride composite phase change materials for solar-thermal-electric energy conversion and storage with very low content of graphene nanoplatelets. Chem. Eng. J. **315**, 481-490 (2017). https://doi.org/10.1016/j.cej.2017.01.045

[S25] J. Yang, P. Yu, L.S. Tang, R.Y. Bao, Z.Y. Liu et al., Hierarchically interconnected porous scaffolds for phase change materials with improved thermal conductivity and efficient solar-to-electric energy conversion. Nanoscale **9**(45), 17704-17709 (2017). https://doi.org/10.1039/C7NR05449A

[S26] J. Yang, L.S. Tang, L. Bai, R.Y. Bao, Z. Liu et al., Photodriven shape-stabilized phase change materials with optimized thermal conductivity by tailoring the microstructure of hierarchically ordered hybrid porous scaffolds. ACS Sustain. Chem. Eng. **6**(5), 6761-6770 (2018). https://doi.org/10.1021/acssuschemeng.8b00565

[S27] L.S. Tang, Y.C. Zhou, L. Zhou, J. Yang, L. Bai et al., Double-layered and shape-stabilized phase change materials with enhanced thermal conduction and reversible thermochromism for solar thermoelectric power generation. Chem. Eng. J. **430**, 132773 (2022). https://doi.org/10.1016/j.cej.2021.132773

[S28] F. Xue, X.Z. Jin, X. Xie, X.D. Qi, J.H. Yang et al., Constructing reduced graphene oxide/boron nitride frameworks in melamine foam towards synthesizing phase change materials applied in thermal management of microelectronic devices. Nanoscale **11**(40), 18691-18701 (2019). https://doi.org/10.1039/C9NR07273J

[S29] Z. Qian, H. Shen, X. Fang, L. Fan, N. Zhao et al., Phase change materials of paraffin in h-BN porous scaffolds with enhanced thermal conductivity and form stability. Energy Build. **158**, 1184-1188 (2018). https://doi.org/10.1016/j.enbuild.2017.11.033

[S30] X. Fang, L.W. Fan, Q. Ding, X.L. Yao, Y.Y. Wu et al., Thermal energy storage performance of paraffin-based composite phase change materials filled with hexagonal boron nitride nanosheets. Energy Convers. Manage. **80**, 103-109 (2014). https://doi.org/10.1016/j.enconman.2014.01.016

[S31] D.G. Atinafu, W. Dong, J. Wang, X. Huang, J. Wang et al., Synthesis and characterization of paraffin/metal organic gel derived porous carbon/boron nitride composite phase change materials for thermal energy storage. Eur. J. Inorg. Chem. **2018**(48), 5167-5175 (2018). https://doi.org/10.1002/ejic.201800811

[S32] Z. Yang, L. Zhou, W. Luo, J. Wan, J. Dai et al., Thermally conductive, dielectric PCM–boron nitride nanosheet composites for efficient electronic system thermal management. Nanoscale **8**(46), 19326-19333 (2016). https://doi.org/10.1039/C6NR07357C

[S33] M. Fashandi, S.N. Leung, Sodium acetate trihydrate-chitin nanowhisker nanocomposites with enhanced phase change performance for thermal energy storage. Sol. Energy Mater Sol. Cells **178**, 259-265 (2018). https://doi.org/10.1016/j.solmat.2018.01.037

[S34] D. Su, Y. Jia, G. Alva, F. Tang, G. Fang, Preparation and thermal properties of n–octadecane/stearic acid eutectic mixtures with hexagonal boron nitride as phase change materials for thermal energy storage. Energy Build. **131**, 35-41 (2016). https://doi.org/10.1016/j.enbuild.2016.09.022
